# Automated weed monitoring and control: enhancing detection accuracy using a YOLOv7-AlexNet fusion network

**DOI:** 10.3389/fpls.2025.1664650

**Published:** 2025-11-25

**Authors:** Muhammad Faizan Zeb, Abid Iqbal, Ghassan Husnain, Wisal Zafar, Ahmad Junaid, Ali Saeed Alzahrani, Syed Hashim Raza Bukhari, Ramasamy Srinivasaga Naidu

**Affiliations:** 1 Department of Computer Science, Iqra National University Peshawar, Peshawar, Pakistan; 2 Department of Computer Engineering, College of Computer Sciences and Information Technology, King Faisal University, AlAhsa, Saudi Arabia; 3 Department of Computer Science, Cecos University of Information Technology (IT) and Emerging Sciences, Peshawar, Pakistan

**Keywords:** deep learning, YOLOv7, AlexNet architecture, precision agriculture, weed detection & classification

## Abstract

The agricultural sector is crucial to global sustainability, but it still faces challenges, particularly from weed invasions that severely compromise crop yields. Although considerable efforts have been made to address the weed problem using computer vision detection methods, the technology is still limited. Weedy sites or their crop hosts share many perceptual features, making it difficult to detect with confidence. Most weed detection methods used today suffer from several problems: the inability to distinguish crops from similar-looking weeds, inconsistent performance across weed growth stages, and sensitivity to operational constraints. Previous methods have employed models such as YOLOv5, ResNet, and Faster R-CNN, but have suffered from issues with accuracy, estimation times, and the ability to detect small weeds in dense stands. In this study, we present a hybrid deep learning system that utilizes YOLOv7 for weed detection and AlexNet for weed species classification. YOLOv7 was used due to its fast recognition capabilities and ability to discriminate with better granularity when detecting grass in dense environments. It was found that using AlexNet to classify weed species accurately increases the specificity of the system. Experimental results of the hybrid model demonstrated improvements over previous methods, achieving a precision, recall, F1 score, mAP@0.50, and mAP@0.5:0.95 of 0.80, 0.85, 0.87 0.89, and 0.50, respectively. The field test detection capability showed that AlexNet achieved precision, recall, and F1 scores of 95%, 97%, and 94%, respectively. Thus, these results indicate that the YOLOv7-AlexNet hybrid model provides both robust and efficient real-time detection and classification of weeds in agriculture. The next step is to expand the dataset to include a wider variety of weed species and environmental conditions, and to validate the developed model by deploying the YOLOv7-AlexNet hybrid model on field computers, thereby expanding its practical application in production environments.

## 1 Introduction

Weed management has always been a significant concern in agriculture, as weeds harm crop production and resource use efficiency. Weeds compete with crops for sunlight, moisture, and nutrients, which reduces the quantity and quality of agricultural production. According to the FAO, weeds are estimated to account for 34% of agricultural losses worldwide, making it imperative that we find more effective or scalable methods of weed control (Atta et al., 2023). As global food demand continues to grow, the need for more sophisticated weed detection technology also increases. Farmers traditionally relied on manual scouting to assess weeds in their fields (Singh et al., 2020). This often involved labor-intensive scouting methods, such as sampling farmers’ crops at predetermined intervals using zigzag patterns or employing crop scouts. Labor costs, time, and human susceptibility to error and bias (Niyigena et al., 2023). Many farmers have attempted to reduce manual labor by using synthetic herbicides; however, this approach has led to other problems, including herbicide resistance, environmental degradation, and soil and water contamination by heavy metals (Hassan et al., 2023).

To tackle these issues, the research uses a hybrid framework based on deep learning that can automatically extract discriminative features across different field conditions, while data augmentation and various training samples improve the model’s robustness to variability in the natural environment. Conversely, they generally did not perform well under field conditions with all the additional inherent random variables that affected the results based on natural environmental factors, such as light, weed density, obstruction, and crop similarities. Furthermore, their generalization was limited by their reliance on manually created features for engineering, which can reduce their effectiveness in many situations across various types of agricultural areas (Bruinsma, 2017).

Deep learning (DL), and especially convolutional neural networks (CNNs), have changed the game of visual recognition tasks (including in precision agriculture). By automatically extracting multi-level features from raw image data, DL-based approaches can eliminate human biases that typically arise from preprocessing steps used to classify agricultural images. All DL-based methods have improved the overall classification accuracy of the original image data. Several different implementations of DL methods (ResNet, VGG, Faster R-CNN, YOLOv3, YOLOv4, and YOLOv5) have been used in weed detection tasks (Saini, 2022; Dargan et al., 2020). As is the case in most studies, existing models still have limitations. Although Faster R-CNN has a high accuracy rate, it remains a two-stage model that requires a second classification stage, resulting in slower inference speeds for images. However, it is more accurate than YOLOv5s (Salazar-Gomez et al., 2021). YOLOv5s, on the other hand, is faster during image inference since it is a single-stage model; however, it has been reported to have COI issues for weed species that are close together, and we were unable to produce solid results for overlapping weed species. Rahman et al., 2023).

To address these constraints, researchers are seeking hybrid models that leverage the strengths of multiple models. In this research, we present a hybrid framework based on deep learning that integrates YOLOv7 and AlexNet to achieve automated weed detection and control. YOLOv7 represents an innovative leap forward in object detection, offering enhanced measures of speed and accuracy through improvements, such as the Extended Efficient Layer Aggregation Network (E-ELAN) (Jiang et al., 2022a). In our framework, YOLOv7 recognizes complex field conditions and identifies weed targets at rapid real-time speeds. Once weed detection is accomplished, the features of the weeds are passed to AlexNet, a powerful CNN framework proposed for efficiency in classification tasks. While the weeds are already classified, AlexNet is used for fine-grained identification of weed species (Beeharry and Bassoo, 2020).

In this research, we develop a novel hybrid framework that combines YOLOv7 and AlexNet to address the primary challenges associated with automatic weed detection and classification in agricultural settings. This framework enables the detection of small and overlapping weeds in real-time and is easily integrated with unmanned aerial vehicles (UAVs) or robots, further advancing by allowing the classification of visually similar species. The proposed architecture is modular and can be scaled to different agricultural settings. It incorporates sustainable agriculture principles by leveraging additional monitoring strategies to minimize reliance on negative herbicides and support data-driven precision agriculture. Specifically, we apply transfer learning, where pre-trained learning models, deceptively trained on large and complex datasets, enable efficient and effective learning (Iman et al., 2023). Combining YOLOv7’s fast and accurate weed detection capabilities with AlexNet’s lightweight yet effective classification offered a unique approach for automated weed detection, localization, and classification (López-Correa et al., 2022). YOLOv7 can effectively detect weeds in complex and crowded field conditions, which is vital for timely action and reduced herbicide use. At the same time, AlexNet provides efficient and accurate species classification at a superior level. The strengths of both modes combine to create a functional, real-world solution for agricultural scenarios, significantly enhancing the efficacy, efficiency, and sustainability of modern weed management systems. This study aims to (1) create an integrated deep learning framework consisting of YOLOv7 and AlexNet for real-time weed detection and classification; (2) assess the model’s detection performance for small, overlapping, and visually mimicking weed species; and (3) analyze the usability of the proposed model for utilization in real-time agricultural contexts (i.e., UAVs or mobile robots). The vital contributions are summarized as follows:

- The authors proposed a hybrid deep learning approach that utilized YOLOv7 for deep learning object detection and AlexNet for classification, enabling real-time and accurate weed sensing within a single system.
- The model improved detection ability, especially when detecting small weeds, overlapping weeds, and closely growing weeds in complex paddock environments. This addressed one of the shortcomings of previous methods.
- By integrating AlexNet, the method demonstrated improved classification ability in detecting weeds belonging to the same genus with similar morphological characteristics.
- The proposed model retained real-time inference times, making it suitable for deployment on UAVs, mobile robots, or other real-time agricultural platforms.
- The authors incorporated modular and scalable architecture, allowing the method to quickly transfer or adapt to different agricultural contexts and datasets with minimal training.
- The suggested system could encourage environmental sustainability in agriculture through enhanced precision and timeliness of weed identification, which may help mitigate excessive reliance on herbicide applications in future practices.
- Transfer learning techniques enhanced the performance and generalizability of the model, enabling effective training with limited quantities of annotated agricultural datasets.

The organization of this paper is as follows: Section 1 outlines key aspects related to agriculture and weed control. A brief overview of the existing work in deep learning and machine learning methods for weed identification is presented in Section 2. Section 3 describes the materials and the experimental methods used for categorization and detection of weeds. Some of the methods involved in this study include feature selection, bounding box selection, weed identification and classification. Section 4 of the paper presents the results, and the discussion of the study. Section 5 outlines the conclusion and future work of the proposed algorithms.

## 2 Literature survey

In the literature review section, the **Table 1** summarizes the comparative analysis of the analyzed methods, describing the methodology, performance information, key limitations, and contributions to the need for a hybrid model. The table provides a clear overview of the research gaps and adequately substantiates the need to propose a better model. The researchers (López-Martínez et al., 2023) reported that the experiment yielded an accuracy of 0.65 for CNN models, utilizing an HPC cluster to classify the weeds. The optimal training time for CNN models in the experiment was 37 minutes and 55.193 seconds, utilizing six HPCC cores. It has been observed that using the Lightweight Deep Learning model, YOLO5 outperforms SSD-ResNet50 in weed identification and classification. It supports commercial real-time weed control through an autonomous laser-weeding robot. In the context of weed detection, the mAP of YOLOv5 was recorded at 0.88 @0.5. The YOLOv5 model achieved a frame per second (FPS) rate of 27. Another research group reported that Deep learning techniques, particularly Transformer models such as SegFormer, have proven highly effective for the detection and classification of weeds. The model offers greater accuracy and efficiency compared to traditional methods. SegFormer achieved a Mean Accuracy (mAcc) of 75.18% and a Mean Intersection over Union (mIoU) of 65.74%. In contrast, Swin Transformer had nearly five times as many parameters compared to SegFormer.

**Table 1:** Literature integrated summary.

Author & ref	Dataset	Method	Classification	Accuracy	Limitation
López-Martínez et al., 2023	DeepWeeds	HPCC infrastructure + DL (CNN)	Multiclass	0.65%	The HPCC requires a high-quality network infrastructure to operate effectively.
Fatima et al., 2023	Weed Images Collected (9000)	YOLO5 + SSD-ResNet50	Multiclass	0.88 (mAP)	The dataset includes only a Limited Number of weed species, with a total of only 4 species used.
Jiang et al., 2022b	Weed (1006) images	Swin Transformer, SegFormer and Segmenter.	Multiclass	75.18%	Limited number of images and only 10 species are used.
Gallo et al., 2023	2 Dataset Lincoln Beet (LB) + Chicory Plant (CP) (4402) images	YOLOv7	Binary	56.6%	The two datasets comprise a total of 4,402 images, with only two classes being used.
Khan et al., 2022	Collected locally not available publicly	Tiny-YOLOv4	Multiclass	49.4% (mAP)	Limited dataset and limited classes. Old Version of Yolo is used.
Liu et al., 2022a	1000 images of maize seedlings and weeds	Multiple YOLOv4-tiny	Multiple	86.69% (mAP)	The training data was imbalanced, and the model was only tested on weeds during the maize seedling stage with a limited Number of Classes.
Saleem et al., 2022	Deep Weeds (17,509), 8 Classes	Faster RCNN	Multiclass	87.64%, (mAP)	Limited number of classes.
Hashemi-Beni et al., 2020	Crop/Weed Field Image 60 Images	U-Net and FCN-8s,	Multiclass	(75.1%) & (66.72%)	There is a need to enhance segmentation and further improve object detection.
Sivakumar et al., 2020	Soybean weed	Faster R-CNN and Single Shot Detector (SSD)	Single Class	0.85 (Mean IoU) 0.84 (Mean IoU)	The dataset exhibited limited variation, and the SSD model showed higher misclassification rates, particularly for the limited Number of Classes.
Kulkarni, 2019	250 Crop and weed images	CNN model	Binary	85%	Small dataset, no specific weed species.
Sarker and Kim, 2019	2000 images.	(R-FCN) with ResNet-101	Multiclass	0.81% (mAp)	A small dataset was used, and dropout techniques and data augmentation were employed to overcome the limitations of the small dataset size.
Sampurno et al., 2024	5000 images Acquired from (T-PIRC)	YOLOv5 and YOLOv8	Multiclass	82.40 (mAp) 82.10 (mAp)	With a limited dataset, the model could be further trained on more diverse data from other orchard environments.
Xu et al., 2024	grass weeds and wheat	WeedsNet	Binary	62.3%	Limited datasets need to enhance the accuracy.
Hashemi-Beni et al., 2022	Crop/Weed Field Image Dataset & Sugar Cane Orthomosaic dataset	SegNet, FCN-32s, FCN-16s, FCN-8s, U-Net	Multiclass	84.3% 81.1% 77.9%.	The precision has to be improved. A little dataset was utilized.
Saini and Nagesh, 2024	CottonWeeds	YOLOv5 models	Multiclass	87.4% (mAp)	Utilized a little dataset. The precision has to be improved.
Rai et al., 2023	common, annotated imagery dataset	YOLOv3 object detection model	Multiclass	54.3% (mAp)	A limited number of images and weed species were used.

The study, evaluated by (Jiang et al., 2022b), assessed the effectiveness of the YOLOv7 model for weed detection using UAV images of chicory plantations and the Lincoln beet dataset. On the Chicory Plant (CP) dataset, YOLOv7 achieved promising results with mAP@0.5 scores, recall, and precision scores of 56.6%, 62.1%, and 61.3%, respectively. When applied to the Lincoln beet (LB) dataset, YOLOv7 outperformed previous models by increasing the mAP@0.5 scores from 51% to 61%, the mAP for weeds from 67.5% to 74.1%, and the mAP for sugar beets from 34.6% to 48%.

The Tiny-YOLOv4 model was employed to detect potato weeds in real-time (Gallo et al., 2023; Khan et al., 2022). On a limited dataset, this model’s testing accuracy was 49.4%. The best performing model was used to identify weeds in potato fields.

The images of corn and weeds were manually collected and labeled in one study (Liu et al., 2022a). Several YOLOv4-tiny network models were trained and evaluated. The real-time detection of weeds in maize seedlings using deep learning, particularly YOLOv4-tiny, achieved a mean Average Precision (mAP) of 86.69%, a detection speed of 57.33 frames per second (f/s), and a model size of 34.08 MB. These results highlight the model’s effectiveness in various weather conditions. The Faster R-CNN architecture achieved the highest mean average accuracy score among all the evaluated models. It was trained using various deep learning backbones and classification models, including Inception-v2, ResNet-50, and ResNet-101. The final model utilized was ResNet-101, which effectively learned the features that distinguish seven weed classes and negative classes. The mAP reached 87.64%, demonstrating effective detection of most weed classes.

The studies proposed by (Saleem et al., 2022; Hashemi-Beni et al., 2020) utilize the deep learning algorithms to categorize remote sensing images for agricultural purposes, with a particular focus on crop and weed identification using the U-net and FCN-8s models. U-Net showcased its capability by achieving an impressive standard accuracy of 72.2%. Meanwhile, FCN-8s experience a significant decrease, but maintain a high accuracy of 72.1%. The effects highlight the use of deep learning techniques, such as U-Net and FCN-8s, to improve crop and weed identification in agricultural settings. Ongoing upgrades and optimization of this system can enhance accuracy and effectiveness benefiting precision agricultural operations.

The researchers (Sivakumar et al., 2020) evaluated weed identification by comparing two object identity models, Faster R-CNN and Single Shot Detector (SSD), using IoU and inference speed. Regarding performance, recall, F1 score, and IoU, the Faster R-CNN version with 200 box suggestions demonstrated overall weed detection performance comparable to that of the SSD model. Notably, the inference time taken by the Faster RCNN model was the same as that of the SSD version. With 200 suggestions, the Faster R-CNN model has achieved an IoU of 0.85, an F1 score of 0.66, a precision of 0.65, and a recall of 0.68. In contrast, the SSD model achieved an IoU of 0.84, an f1 score of 0.67, an Accuracy of 0.66 and a Recall of 0.66. The recognition accuracy was higher for the optimal confidence threshold of the SSD, but it lagged behind the Faster R-CNN model. The SSD requires improved generalization capability for utilizing UAV data to detect weeds in the mid-to-late stages of soybean fields. IoT-based weed detection systems utilize CNN and image processing for classifying weeds, allowing for remote monitoring of crops and reducing the need for manual labor and chemical usage in agriculture. The system achieves an average accuracy of 85% with 250 training images, along with an average false ratio of 7% and a false acceptance ratio of 2.6%.

The researchers (Kulkarni, 2019; Sarker and Kim, 2019), implemented Region-based deep convolutional neural networks, particularly ResNet-101 with R-FCN, demonstrate exceptional performance in detecting weeds in farmland. They show better performance in object classification performance supported by dropout techniques and data augmentation. ResNet-101 with R-FCN outperforms other methods in object detection. The implementation of data augmentation and dropout techniques effectively reduces overfitting. Our proposed method achieves an accuracy of 81%, comparable to the baseline set by both the Faster R-CNN and R-FCN. It performs especially well with datasets and improves object identification accuracy.

This study proposed by (Sampurno et al., 2024) to evaluate each YOLO model based on detection accuracy, model complexity, and inference time conducted a comparative analysis. They observed that smaller variants, such as YOLOv5 and YOLOv8, have proven more effective than their larger counterparts. For detection, YOLOv5n-seg achieved an mAP@0.5 value of 80.90%, while YOLOv8s-seg achieved a value of 82.40%. a model for detecting weeds in wheat fields using RGB images is challenging due to the similar appearance of grass weeds and wheat (Xu et al., 2024). A dual-path weed detection network (WeedsNet) has been proposed to address this issue by utilizing both RGB and depth images. WeedsNet effectively combines multi-modal information, significantly enhancing detection accuracy to 62.3% in natural wheat fields, while achieving a detection speed of 0.5 seconds per image. Precise crop/weed mapping is essential for targeted treatment, and advancements in UAS-based remote sensing and deep learning have improved this process. This research compares various deep learning methods for crop/weed discrimination using UAS data. It evaluates several U-Net, SegNet, FCN (32s, 16s, 8s), and DeepLabV3+ architectures. The impressive performance, utilizing the resources of DeepLabV3+, was highlighted by its accuracy of 84.3%, with FCN-8s at 81.1% and U-Net at 77.9%. These results indicate the significant potential of the ResNet-18-based DeepLabV3+ to eliminate weeds.

Another research group utilizes pre-trained YOLOv5 models for weed identification, with YOLOv5x achieving remarkable results, including a mAP_0.5:0.95 score of 72.5% and a mAP_0.5 score of 87.4% (Hashemi-Beni et al., 2022; Saini and Nagesh, 2024). The result indicates that the model is effective in identifying weeds in cotton fields. The research demonstrates the potential of CottonWeeds as a key training platform for developing real-time, in-field weed recognition systems.

The successful implementation of site-specific weed management (SSWM) requires the reliable identification of weed species, as well as their exact location on the site (Rai et al., 2023). The YOLOv3 model effectively identified multiple weeds of different species, achieving precision scores of 43.28%, 26.30%, 89.89%, and 57.80% for the possessed species, and a mAP of 54.3%. These findings highlight the potential of deep learning models for weed identification and the benefits of object detection for SSWM.

Contemporary deep learning networks, such as ResNet and VGG, can identify objects of interest; however, these models come with very high computational costs, making real-time object detection impractical. YOLO models, particularly YOLOv3, YOLOv4, and YOLOv5, offer faster weed detection. Nevertheless, their accuracy diminishes when weeds are small or tightly placed. This limitation leads to the selection of YOLOv7 due to its enhanced properties.

## 3 Material and techniques

**Figure 1:**
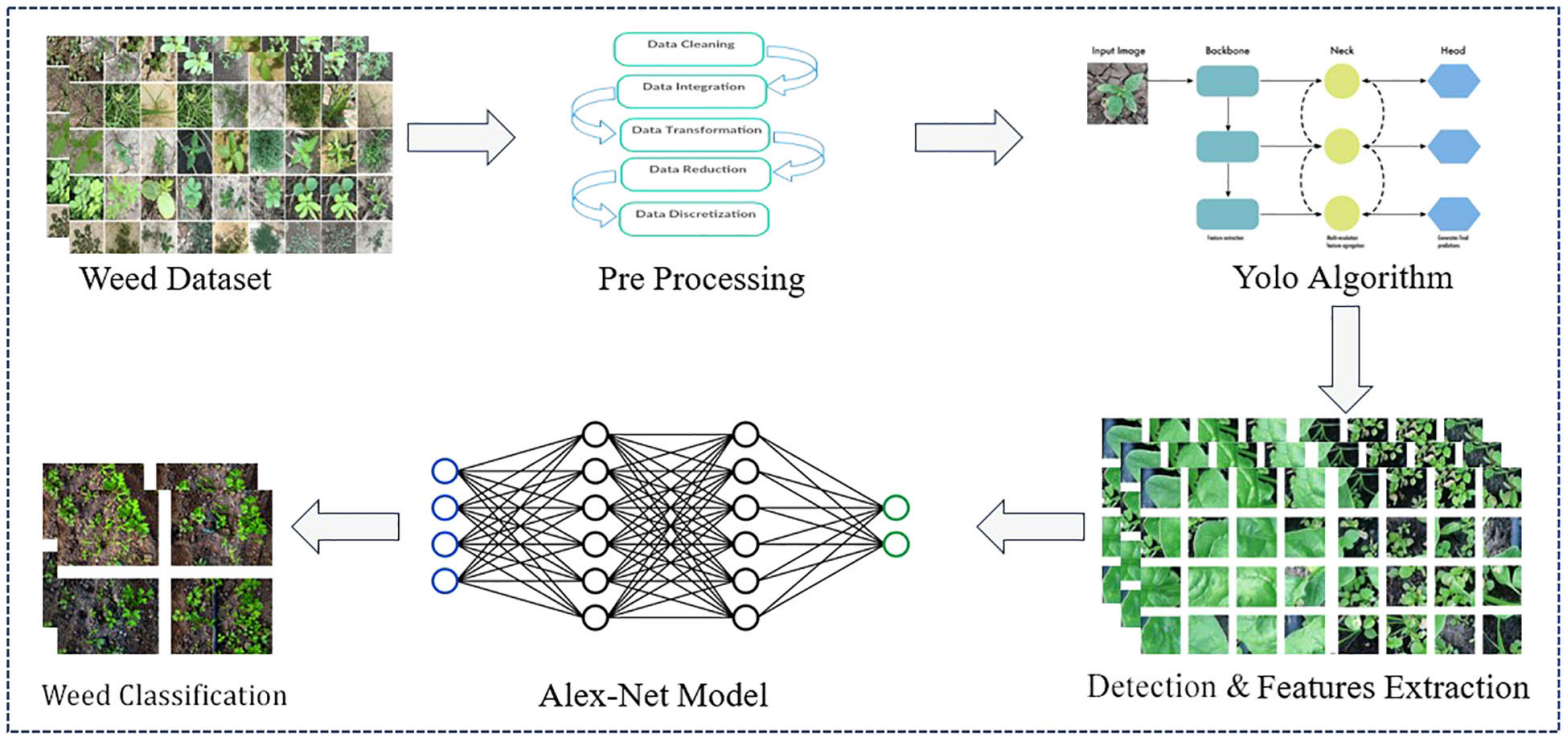
Hybrid proposed model for weed detection and classification.

The process in the diagram integrates YOLOv7 and AlexNet deep neural network systems, where weed detection is performed using YOLOv7, and its classification is determined by AlexNet, as illustrated in **Figure 1** below. Here is a thorough, step-by-step breakdown:

### 3.1 Brief introduction of the hybrid proposed for weed detection and classification

#### 3.1.1 Proposed YOLOv7-AlexNet methodology

The proposed framework employs a hybrid architecture that combines YOLOv7 and AlexNet to rapidly and reliably detect and classify weeds in the field. **Figure 1** depicts the initial labeled weed image dataset to input into the model, followed by the preprocessing steps, which include data cleaning, data transformation, and data normalization to enhance the consistency of the input data and the model’s performance.

The final preprocessed images would then be passed through the detection module based on YOLOv7, which would determine the weed’s location in real-time by creating spatial bounding boxes. YOLOv7 was selected in this framework due to its speed and ability to detect small weeds, overlapping weeds, or weeds growing in clumps, similar to how they would appear in a varied agricultural field environment. The weed-localized areas would then be passed to the classification module based on the AlexNet architecture, which provides specific species-level class labels for the weed, such as foxtail and thistle. The AlexNet model is lightweight and capable of classifying images rapidly with an acceptable degree of accuracy, enabling real-time and embedded applications in agricultural settings. The underlying principles behind the model’s evaluation performances depend on an implicit feature fusion strategy. YOLO outsources its goal detection in space, while AlexNet performs an evaluation of the image and looks for visual similarities with its previous identification (Murad et al., 2023). The intermittent system benefits from increased robustness and generalization across different agricultural problem stations.

The end product merges YOLOv7 localization with AlexNet’s classification to develop annotated images that combine bounding boxes and species names. This enables practical applications on UAVs or robotic vehicles for precision weed management and can also facilitate real-time decision-making for sustainable farming practices.

For our classification framework, we adopted a hybrid architecture combining YOLOv7 for object detection and AlexNet for final classification. Individually, AlexNet offers fast inference (~20 ms) and low power consumption (~150 W), but with moderate classification accuracy (~73%). In contrast, YOLOv7 provides significantly higher accuracy (up to 99.4%) and faster detection (~14 ms), though at a higher computational cost. By integrating YOLOv7’s precise localization with AlexNet’s lightweight classification, the hybrid model achieves a balanced performance, reaching an overall accuracy of ~99.46% with a total inference time of ~34ms. This approach ensures high efficiency and accuracy, making it well-suited for real-time applications in resource-constrained environments (Ndlovu et al., 2020).

#### 3.1.2 Detection and feature extraction

The YOLOv7 algorithm performs the detection process by recognizing and extracting the spatial and contextual features of weeds from the input images. These types of features include significant attribute details, such as weed shape, size, and position. The detection is performed using YOLOv7’s architecture, which comprises a backbone for extracting features, a neck to collect features across scales, and a head for object detection and bounding box prediction. Next, the features extracted, which include much more than bounding box coordinates, will contain high-dimensional, enriched intermediate representations that are ultimately used as input for the AlexNet model. AlexNet, a convolutional neural network architecture for image classification, processes the images through its different layers to evolve the representation of weeds into class-specific representations. Once these features are passed through AlexNet, weeds are classified into specific weed species (Pandey et al., 2022).

It is essential to note that the models YOLOv7 and AlexNet are trained sequentially, rather than jointly. YOLOv7 is first trained to detect weeds accurately, and then the dataset of inputs, as feature outputs from YOLOv7, is used to fine-tune AlexNet for the classification of weed species. The sequential approach to training the models enables the weed detection capability unique to YOLOv7 to evolve classification as a function of AlexNet’s generic architecture. In contrast to a more complex joint approach, this approach minimizes residual confusion in detection relative to classification by maintaining a clear transition from detection to classification while still developing a tidy model integration pipeline.

#### 3.1.3 AlexNet model

After YOLOv7 completes the detection process, the next step involves classifying the identified weed objects in detail using the AlexNet model. Instead of taking an image as input, AlexNet takes the feature representation produced by YOLOv7. The features produced by YOLOv7 are high-level features that include spatial and contextual aspects of the weed objects; therefore, they should provide input to help the model better distinguish between weed species that share particularly close visual similarities.

AlexNet is a convolutional neural network specifically designed for classification tasks. It is designed to perform classification using a hierarchy of convolutional layers that aim to extract and recognize complex hierarchical patterns important for distinguishing between visually similar weeds. Pooling layers have the role of reducing dimensionality while preserving the more discriminative features. Certain activation functions, such as ReLU, can allow non-linearity and promote deeper and more abstract learning (Omilola and Robele, 2017). Once all the features have been processed, layers of fully connected neurons aggregate the learned features and produce class probabilities for the final classification. This enables the accurate discrimination of weed species after identifying and localizing the weed objects. The two networks were trained sequentially: YOLOv7 was first trained with optimization for localization, and then its output features were used to train and optimize AlexNet for classification in a standalone manner. Allowing the two models to be learned independently of each other enables each model to specialize in its respective activity, thereby increasing the robustness and interpretability of the entire system.

### 3.2 Configuring the hyper parameters and fine-tuning the hybrid model

#### 3.2.1 Fine tuning

The hybrid model proposed utilizes transfer learning by successively fine-tuning two pre-trained models: YOLOv7 as a weed detector and AlexNet as a weed classifier. The first training phase involves YOLOv7, which is trained independently to detect and localize weeds in agricultural input images. After YOLOv7 is trained, the intermediate feature vectors are extracted from YOLOv7 and then used as input to fine-tune the AlexNet model, which recognizes them as “input” features for species-level classification. Fine-tuning is accomplished in the following ways to adapt the two models to fundamentally different tasks.

- Layer freezing: The lower layers of both networks (YOLOv7 and AlexNet) are frozen, allowing us to retain the learned low-level features within their respective models. The higher layers of the models are then trained with the given dataset. Layer freezing is a method for adapting specific domain knowledge to the respective models without compromising the general visual features learned from larger datasets.
- Learning rate modulation: During fine-tuning, we can gradually reduce the learning rate to adapt the training to the solution and stabilize each fine-tuning process. Keeping the training modulations small prevents excessive adjustment to the pre-trained weights, which would be beneficial at the beginning (making it easier for the model to learn features relevant to the grass classification domain).
- Regularization method: To limit overfitting, improve generalization, and support performance across a variety of environmental conditions and different types of weeds, dropout and weight loss (L2 regularization) methods are integrated into both model fine-tuning processes.

#### 3.2.2 Hyper parameter for training

We carefully tuned the hyper parameters for our hybrid model, which combines YOLOv7 for weed detection and AlexNet for classification, to enhance overall performance. A learning rate of 0.001 was selected to balance convergence speed and stability during training. The batch size was set to 16, which allowed the model to capture finer details in the dataset without overwhelming computational resources. We conducted training over 100 epochs to ensure optimal performance without overfitting. The following are important hyper parameters for the hybrid model’s training:

- Batch Size: The number of samples processed before the model is retrained or updated is normal. The batch size can range from 16 to 32.
- Epochs: The whole training dataset has been repeated a precise number of times, as defined by the training process. The model is trained for a suitable number of epochs (e.g., 100–200 epochs) until it achieves the best results.

#### 3.2.3 Dataset collection

The first step in this methodology involves gathering a comprehensive dataset of images that depict crops in various conditions, with some images containing weeds and others not. The weed25 dataset in this hybrid model is publicly available on the Baidu search engine (Wang P. et al., 2022). The image resources for Weed25 dataset were gathered from fields and lawns in Chongqing, China, capturing 25 prevalent weed species in East Asia. This dataset specifically includes images of weeds with a balanced class distribution, enhancing the model’s ability to differentiate effectively between various species. **Table 2** describes the Weed25 dataset, comprising 14,023 images categorized into 25 distinct classes. The Weed25 dataset includes a variety of weed species, encompassing Barnyard grass (Echinochloa crus-galli), Crabgrass (Digitaria sanguinalis), Green foxtail (Setaria viridis), Sedge (Cyperus rotundus), Horseweed (Conyza Canadensis), Field thistle (Cirsium arvense), Cocklebur (Xanthium strumarium), Indian aster (Kalimeris indica), Bidens (Bidens pilosa), Ceylon spinach (Basella alba), Billygoat weed (Ageratum conyzoides), White smartweed (Persicaria alba), Asiatic smartweed (Persicaria chinensis), Chinese knotweed (Polygonum chinense), Alligatorweed (Alternanthera philoxeroides), Pigweed (Amaranthus retroflexus), Shepherd’s purse (Capsella bursa-pastoris), Purslane (Portulaca oleracea), Common dayflower (Commelina communis), Goosefoots (Chenopodium album), Plantain (Plantago major), Viola (Viola odorata), Black nightshade (Solanum nigrum), Mock strawberry (Duchesnea indica), and Velvetleaf (Abutilon theophrasti).

**Table 2:** Dataset with total number of images and categories.

Dataset	Total number of images	Number of categories
Weed 25	14,023	25

**Figure 2:**
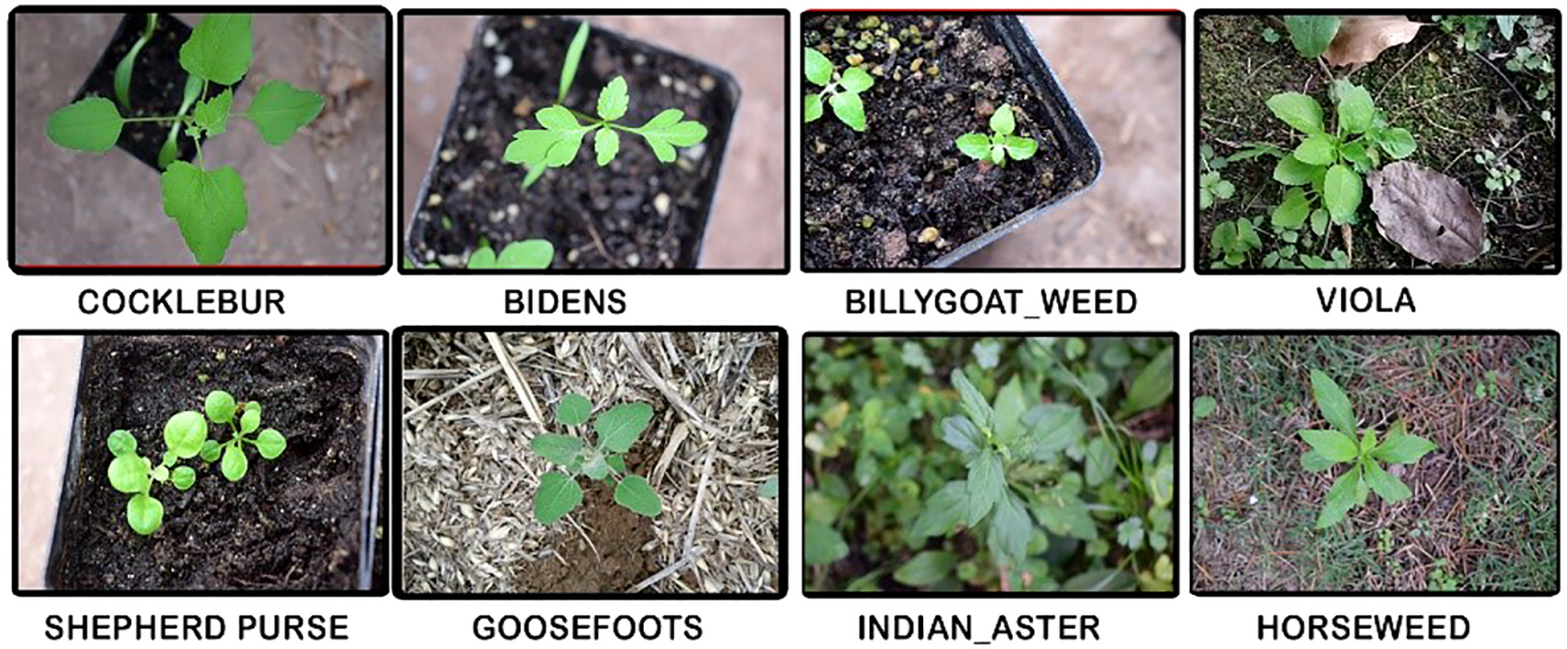
Example of a dataset.

The photos were collected from October 2021 to August 2022. They were captured at a shooting height of around 30 to 50 cm and an angle of 60° to 90°, using a digital camera (Nikon D5300 SLR, Japan) or a smartphone (Huawei Enjoy 9S, China), which ensured a nearly vertical viewpoint of the weeds. To account for the effects of sunlight intensity and angle on weed identification, images were taken between 9:00 and 17:00 under sunny, cloudy, and rainy days, mirroring natural environments. Challenges such as occlusion and overlapping leaves were addressed during image collection. Additionally, some weed species were greenhouse-grown to capture images at various growth stages, primarily between the two- to nine-leaf stages (BBCH 12–19). These diverse conditions were incorporated to prevent the model from overfitting; the model can then be applied to real-life farming practices with new unseen data. Some of the weed25 images are shown in **Figure 2**.

### 3.3 Preprocessing data

The preprocessing step in the methodology is crucial for the dataset to ensure optimal processing and performance by the YOLOv7 algorithm. One important step is image resizing, which involves scaling the photos to a format of40 x 640 pixels for YOLOv7 (Wang et al., 2024). This specific resolution maintains sufficient detail for accurate object identification. This specific resolution provides sufficient detail for accurate object identification while minimizing the analysis load. The size of 640x640 for the images is calculated to be large enough to preserve key object features while remaining small enough for quicker processing and training. Additionally, data augmentation involves applying small, random transformations to the original images, which helps the model become more robust to variations it might encounter in real-world applications. Random rotations were applied to the images, ranging from -15 to +15 degrees. This technique mimics the natural variability in how weeds appear in the field, accounting for different angles and perspectives that might otherwise confuse the model.

### 3.4 Network architecture of YOLOv7

The creators of YOLOv4 and YOLOR created and publicly published YOLOv7 (Wang et al., 2023) in July 2022, marking a significant advancement in the efficiency of object detectors, YOLOv7 surpassed all previous detectors in both speed and accuracy, achieving a remarkable increase from 5 frames per second (fps) to 160 fps This version introduces several architectural changes and a sequence of bag-of-freebies to improve the model’s precision without distending its inferential speed despite considerable augmentation of its training duration (Xing et al., 2023). Two architectural innovations introduced in YOLOv7 are the model scaling for concatenation-based models and the Extended Efficient Layer Aggregation Network (E-ELAN). EELAN is a strategy to improve DML and convergence, utilizing the shortest and most extended gradient path. This approach allows YOLOv7 to stack an unlimited number of computational blocks with various groups that share features through a technique known as feature shuffling, as well as merging cardinality (Terven et al., 2023). This design not only enhances the network’s learning capability but also maintains the integrity of the gradient path. E-ELAN modifies only the computational block while keeping the transition layer architecture intact. Model Scaling involves scaling methods, such as depth scaling, that change the proportions of the input to output channels of the transition layer, which can decrease the hardware’s effectiveness. To this end, YOLOv7 scales the depth and width of the block by the same scale factor as suggested for concatenation-based architectures, thereby maintaining the model’s optimal structure.

As an advanced convolutional neural network, the YOLOv7 architecture, detailed in **Table 3**, is specifically designed for tasks such as weed identification and classification in agricultural contexts.

**Table 3:** Layer details and parameters of the YOLOv7.

Layer	From	N	Params	Module	Arguments
0	–	1	1928	Conv	[3, 32, 3, 1]
1	–	1	118,560	Conv	[32, 64, 3, 2]
2	–	1	19,408	Bottleneck	[64, 64]
3	–	1	174,688	Conv	[64, 128, 3, 2]
4	–	1	3115,200	BottleneckCSP	[128, 128, 3]
5	–	1	1296,448	Conv	[128, 256, 3, 2]
6	–	1	9625,152	BottleneckCSP	[256, 256, 9]
7	–	1	11,181,184	Conv	[256, 512, 3, 2]
8	–	1	32,296,320	BottleneckCSP	[512, 512, 3]
9	–	1	14,722,176	Conv	[512, 1024, 3, 2]
10	–	1	12,101,248	SPP	[1024, 1024, 5, 3, 1]
11	–	1	11,963,008	BottleneckCSP	[1024, 1024, 1]
12	–	1	11,025,024	Conv	[1024, 512, 1, 1]
13	–	1	0	Upsample	[None, 2, ‘nearest’]
14	-1	8	0	Concat	[1]
15	–	1	11,118,208	BottleneckCSP	[1024, 512, 1, False]
16	–	1	1262,656	Conv	[512, 256, 1, 1]
17	–	1	0	Upsample	[None, 2, ‘nearest’]
18	-1	6	0	Concat	[1]
19	–	1	1279,552	BottleneckCSP	[512, 256, 1, False]
20	–	1	131,584	Conv	[256, 128, 1, 1]
21	–	1	0	Upsample	[None, 2, ‘nearest’]
22	-1	4	0	Concat	[1]
23	–	1	156,320	BottleneckCSP	[256, 128, 1, False]
24	–	1	147,712	Conv	[128, 128, 3, 2]
25	-1	20	0	Concat	[1]
26	–	1	313,856	BottleneckCSP	[256, 256, 1, False]
27	–	1	590,336	Conv	[256, 256, 3, 2]
28	-1	16	0	Concat	[1]
29	–	1	1,238,016	BottleneckCSP	[512, 512, 1, False]
30	[23, 26, 29]	12	2,106,822	Detect	[2, [128, 256, 512]]

### 3.5 Backbone (feature extraction)

The backbone of YOLOv7 is designed for feature extraction from images at different scales. Its function can be described as follows:


y=Mish(BatchNorm(Conv(x)))

Where,

- Applying the convolution process to the input x is known as Conv(x).
- BatchNorm: BatchNorm symbolizes Batch Normalization.
- Mish: Mish is the activation function used in YOLOv7, which is a smooth, non-monotonic function.
- Stem Layer: The initial convolutional layers of the network that process the input images in their raw form.
- Stages: A series of convolutional layers that extract features at varying levels of abstraction.
- Cross-Stage Partial (CSP) Layers: To reduce the computational cost and address the vanishing gradient problem, split and combine the feature map after processing.
- SPP (Spatial Pyramid Pooling) Layer: Combines features at various scales while preserving crucial spatial information.

### 3.6 Neck (aggregation and refinement of features)

The neck’s role is to refine and aggregate features from the backbone, preparing them for object detection. The neck’s fundamental structure in YOLOv7 may be described as follows:


y=PANet(x)

Where,

- Ox represents the input feature maps from the backbone.
- PANet(x): A network for path aggregation that maximizes the combination of characteristics from several levels.

### 3.7 Head (detection)

- YOLOv7’s head module makes predictions based on the characteristics that have been analyzed. Its structure is defined as:

y=Sigmoid(Conv(f))

Where,

- f: represent the input feature map coming from the neck.
- Conv: Convolution layers that prepare the features for prediction.
- Sigmoid: Applies the sigmoid function to the predictions for bounding boxes and class probabilities.

### 3.8 Steps of process and results

- Input: An image of a weed is first introduced to the network with standard dimensions of 640 × 640. In YOLOv7 models, one repeated preprocessing step is resizing the image data to a standard size.
- Feature Extraction: The backbone network extracts features using convolutional layers.
- Feature Aggregation: The neck component combines features from different scales.
- Prediction: Bounding boxes, objectness ratings (confidence scores indicating the presence of weeds), and sophistication possibilities are expected to be generated using the head network.
- Post-Processing: Non-most suppression and thresholding are widely engaged to improve detection accuracy.
- Output: Bounding boxes and labels for detected weeds are incorporated in the final output image.

### 3.9 Alex-Net architecture

**Figure 3:**
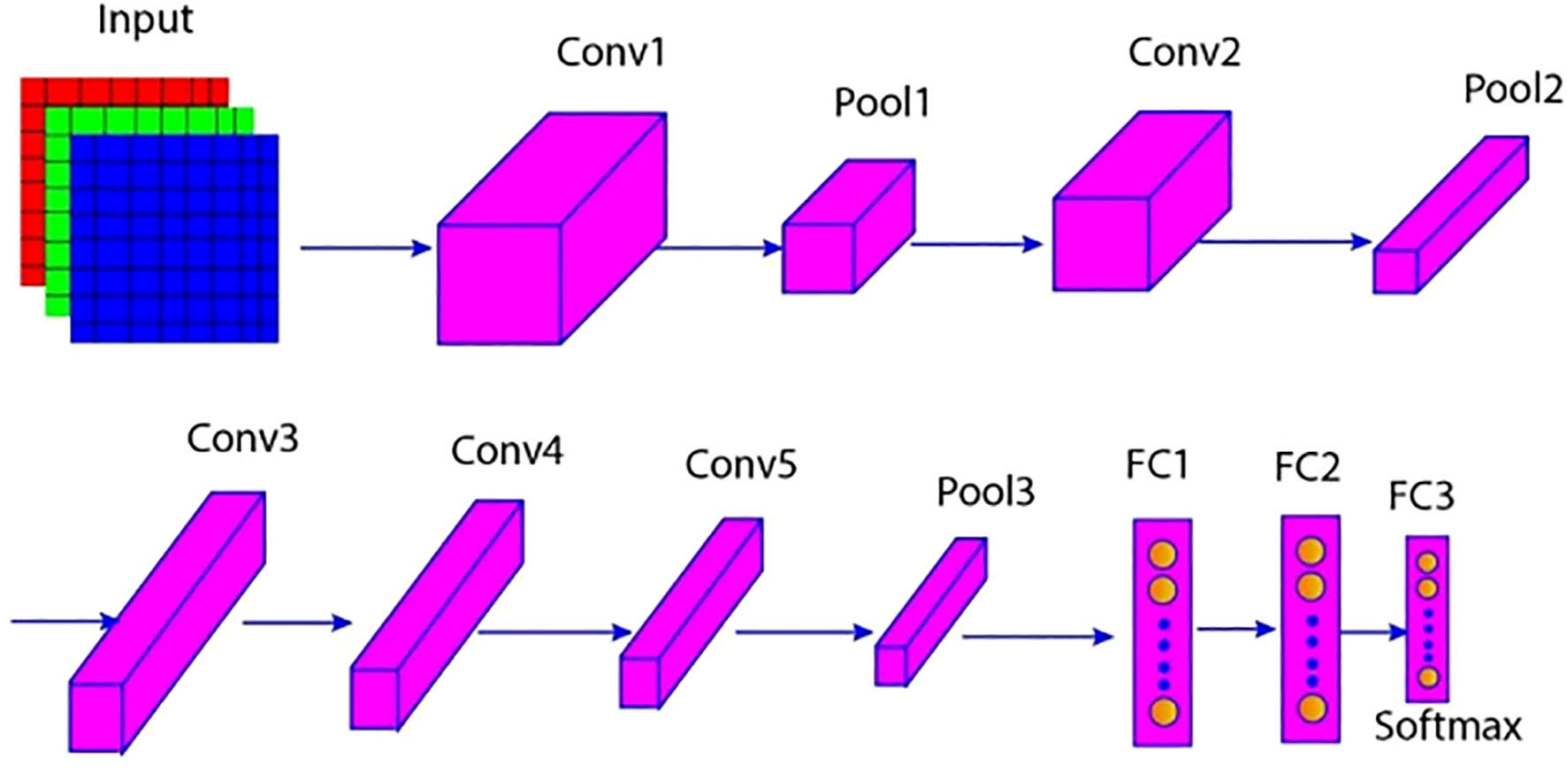
AlexNet architecture.

After identifying and detecting weeds using YOLOv7, the next step is to categorize the types of weeds using an AlexNet model. The extracted features comprise high-dimensional representations from which the vital characteristics and patterns of the input images were obtained before being fed into AlexNet. AlexNet is a convolutional neural network (CNN) architecture commonly used for image categorization tasks (Gikunda and Jouandeau, 2019). Its design enables it to learn details of complex patterns across large datasets, which is helpful for tasks such as weed categorization. AlexNet is composed of three fully connected layers, five convolution layers, and one SoftMax output layer (Patel, 2020). The output aims to identify the probability that the image falls under any of the 1,000 object categories, given the input, an RGB image of size 227 × 227 × 3. The following diagram is marked as **Figure 3**, shows the several levels in the architecture of the sought AlexNet.

#### 3.9.1 Convolutional layers (Conv1-Conv5)

AlexNet’s architecture consists of five convolutional layers that utilize filters to traverse images and identify features (Aloysius and Geetha, 2017). The first layer in the convolutional process uses an 11 × 11 filter, where the features extracted are general. The main layers include the texture and the edges within the images. In the following convolutional layers, the filter size is reduced to 5×5 or 3×3, the learning rate is adjusted, or the model is downsized, allowing these layers to focus on finer details within the significant attributes.

#### 3.9.2 Pooling layers (Pool1-Pool3)

Pooling layers, particularly Max pooling, are vital for convolutional neural networks, such as AlexNet. Their primary function is to sequentially reduce the spatial dimensions of the input data, thereby lowering the network’s computational requirements and the number of parameters (Dhillon and Verma, 2020). At the same time, they help retain the most critical features detected by the convolutional layers.

#### 3.9.3 Normalization layer (Norm1 & Norm2)

In the AlexNet architecture, two Local Response Normalization (LRN) layers normalize the outputs after the first and second convolutional layers. These normalization layers enhance the network’s ability to learn by amplifying important features in the images and suppressing less relevant activations.

#### 3.9.4 Fully connected layers (FC1 – FC3)

AlexNet incorporates three consecutive fully connected dense layers to accurately learn high-level functions from the output of the prior convolutional or max-pooling layers. The first two dense layers include 4096 neurons, while the third and final dense layer includes 100 neurons that correspond with the 1000 classes of ImageNet.

### 3.10 Weed classification

When viewing images of crop fields captured by the system, the YOLOv7 model processes them, defining the various detected regions as bounding boxes containing weeds with probability scores for the level of confidence in instance detection (Pai et al., 2024). For weed localization, it efficiently samples the image space to obtain spatial and contextual feature components. The detected regions will be used in the AlexNet classifier for accurate identification of weed species. AlexNet is a convolutional neural network (CNN) architecture for image categorization that extracts high-dimensional features, possessing general feature extraction capabilities that enable it to distinguish between visually similar features among different types of weeds. More reliable classifications will enable more accurate disease management decisions, resulting in improved weed management performance (Getachew, 2024). The combination of YOLOv7 for weed detection and AlexNet for species classification results in a functional and accurate system for automated weed identification.

### 3.11 System evaluation

The effectiveness of deep learning techniques in weed identification and classification tasks was demonstrated using various overall performance assessment criteria. It is essential to evaluate the efficacy of these methods by focusing on specific performance metrics. In this context, the metrics used to evaluate the performance of the proposed deep learning model include the confusion matrix and related parameters. There is a measure called mean average precision, which evaluates the efficacy of object identification and segmentation algorithms (Padilla et al., 2021). After averaging the average precision (AP) for each class across several training runs, the mean average precision (mAP) is calculated.


$$mAp=\;\frac{\sum_{q=1}^{Q}AveP\;\left(q\right)\;\;}{Q}$$

A confusion matrix is essential for evaluating machine learning models, particularly in classification tasks. It visually represents how the model performs by comparing its predicted outputs to the actual values (Rainio et al., 2024). In Matrix True Positives (TP) are instances where both the observed and expected categories are positive. True Negative (TN) choice values can be linked with situations where the actual and expected categories are harmful. Accuracy is defined as the percentage of correct predictions (including both true positives and true negatives) out of the total predictions made by the model. Recall, also known as sensitivity or the true positive rate, is the ratio of actual positive observations that the model detects to the total number of true positives. Precision, also known as positive predictive value, is the percentage of accurate expected positives. The F1 Score, the harmonic mean of accuracy and recall, is a metric that balances the trade-off between false positives and false negatives.


$$Accuracy=\;\frac{TP+TN}{TP+TN+FP+FN}$$

$$Recall=\;\frac{TN}{TP+FN}\;$$

$$Precision=\;\frac{TP}{TP+FP}\;$$

$$F1\;score=2\frac{PR*RE}{\left(PR+RE\right)}\;$$

## 4 Results and discussion

### 4.1 Overall performance of the model

The YOLOv7 and AlexNet integration was tested on Google Colaboratory (Colab) for computation, utilizing the facility of an NVIDIA Tesla T4 GPU that features seven CUDA cores and 16 GB of RAM. The dataset was split into a 10% test set, a 90% training set, and a 20% validation set, with the split being 70% training and 30% validation. Colab is an open Google platform for data analysis, machine learning, and education. The testing phase evaluates the model’s appropriateness based on unseen data.

### 4.2 Observation and improvement of model results

#### 4.2.1 Yolov7 model’s outcome for weed identification

**Figure 4:**
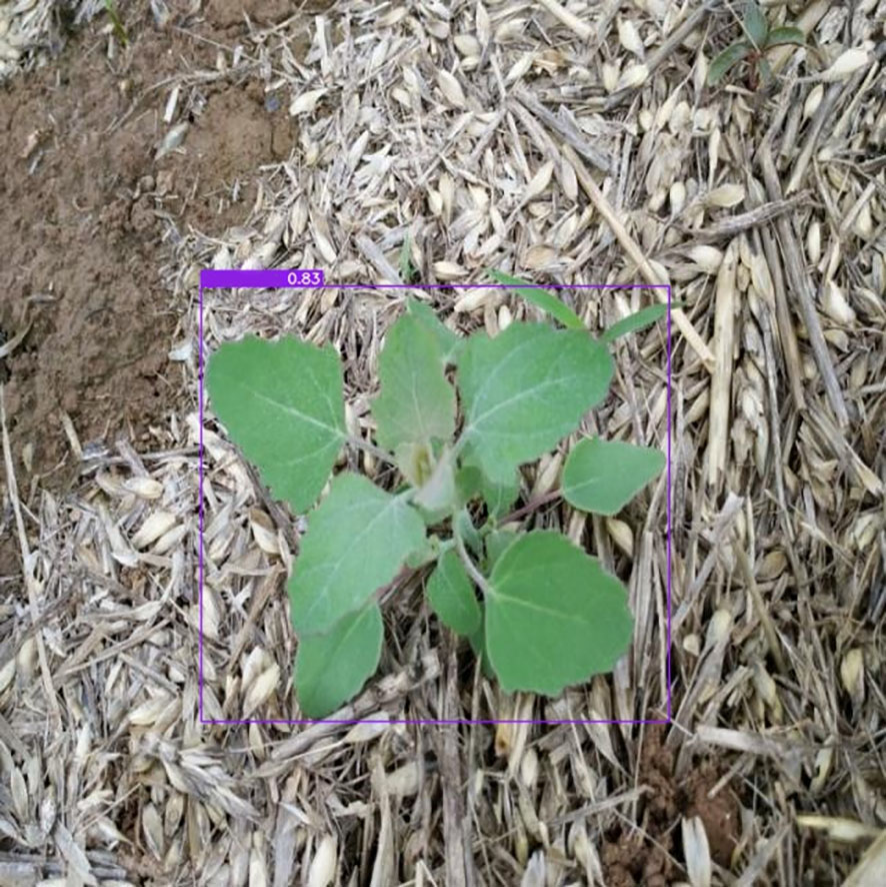
Result of weed detection.

A purple rectangle highlights the detected weed, clearly indicating its location in the image. The text displayed above the picture states that the model is 83% confident in its identification. This high confidence level demonstrates the model’s effectiveness in recognizing weeds, as shown in **Figure 4**.

**Figure 5:**
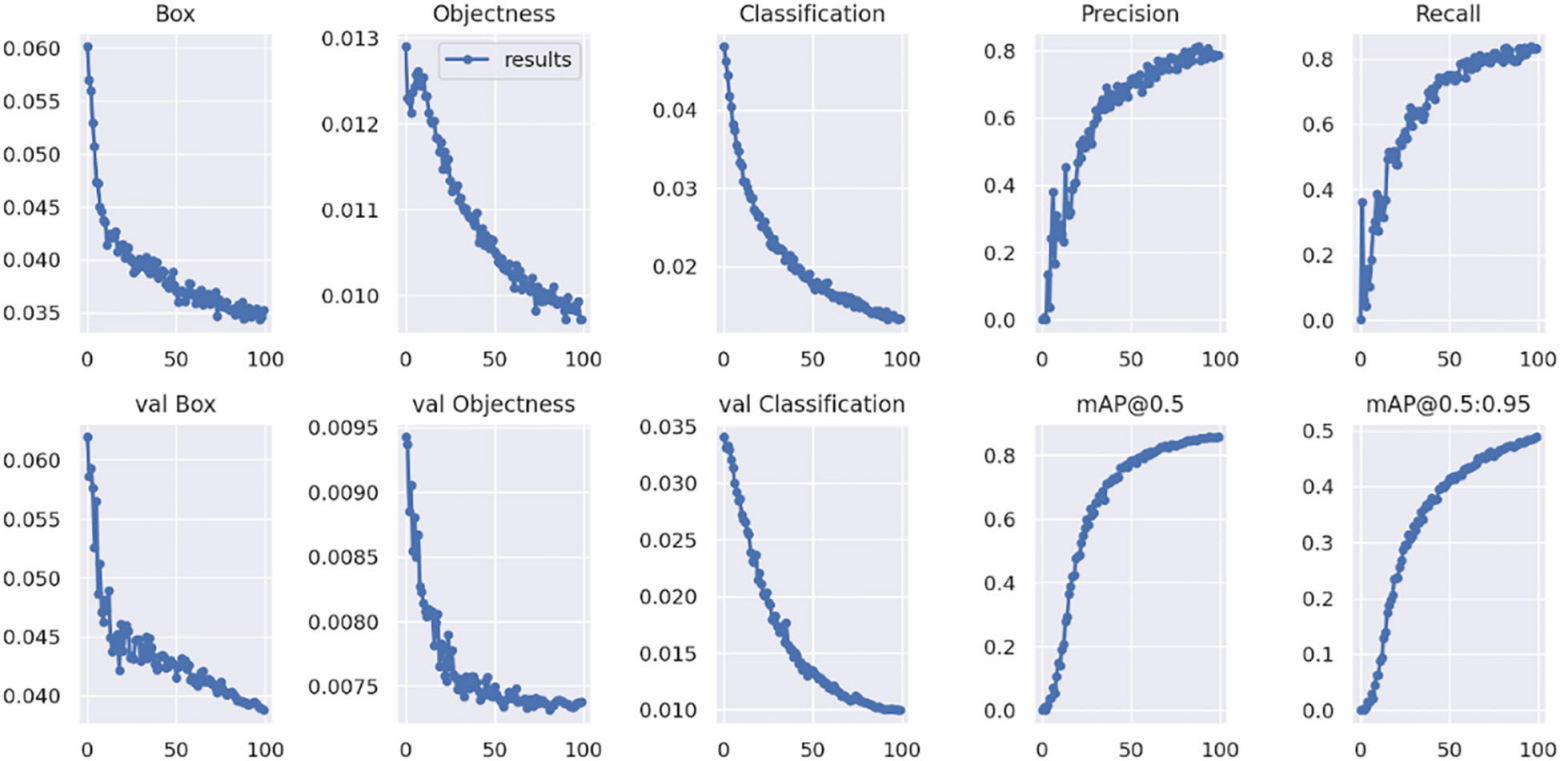
The experimental analysis results YOLOv7 weed detection.

**Figure 5** presents the performance data of the YOLOv7 weed detection model. During the training phase, both box loss and classification loss decrease linearly, indicating an improvement in accuracy for identifying weed plants. The Object loss also declines significantly, allowing for a clear distinction between the weed and its background. In validation, losses become static, demonstrating the use of learned data and proving the model’s existence. Metrics such as precision, recall, mAP@0.5, and mAP@0.5:0.95 remain at high levels, further validating YOLOv7’s capability in accurately identifying weeds. This accuracy is crucial for effective agricultural management and weed suppression.

#### 4.2.2 Result achieved by AlexNet model for weed classification

**Figure 6:**
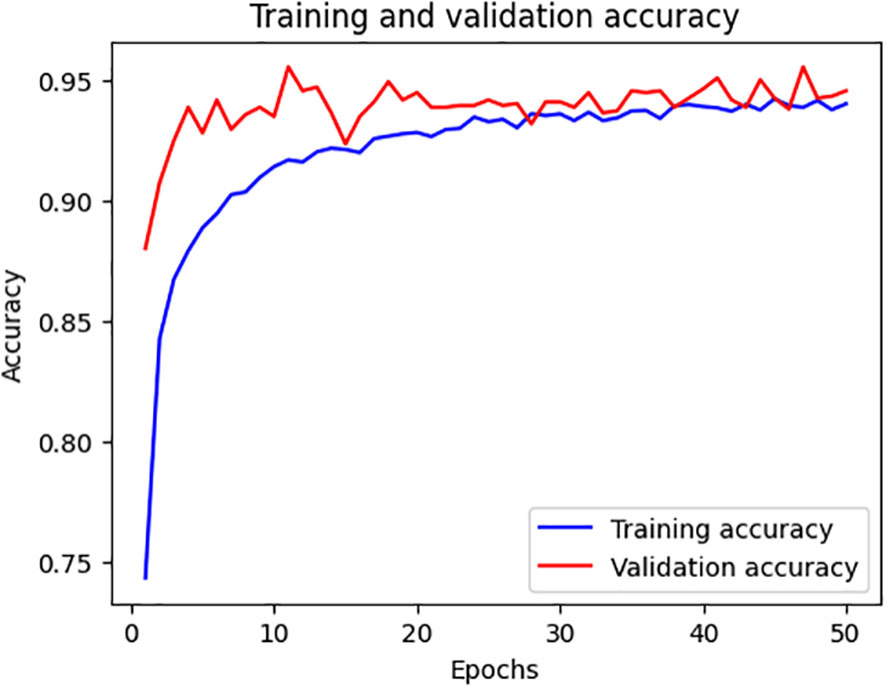
Accuracy chart for the classification of weeds.

The model is performing well, as demonstrated by the training and validation accuracy presented in **Figure 6**. A high validation accuracy at the beginning indicates the proper selection of architecture and hyperparameters. The accuracy begins to stabilize around 30 epochs, indicating the model’s consistency. Experiments show only minor improvements in the test set validation accuracy, suggesting limited overfitting. Both accuracies are kept higher than 0. 90, thus confirming the model’s reliability and consistency in weed detection in research studies.

#### 4.2.3 Misclassification analysis and mitigation strategies

**Figure 7:**
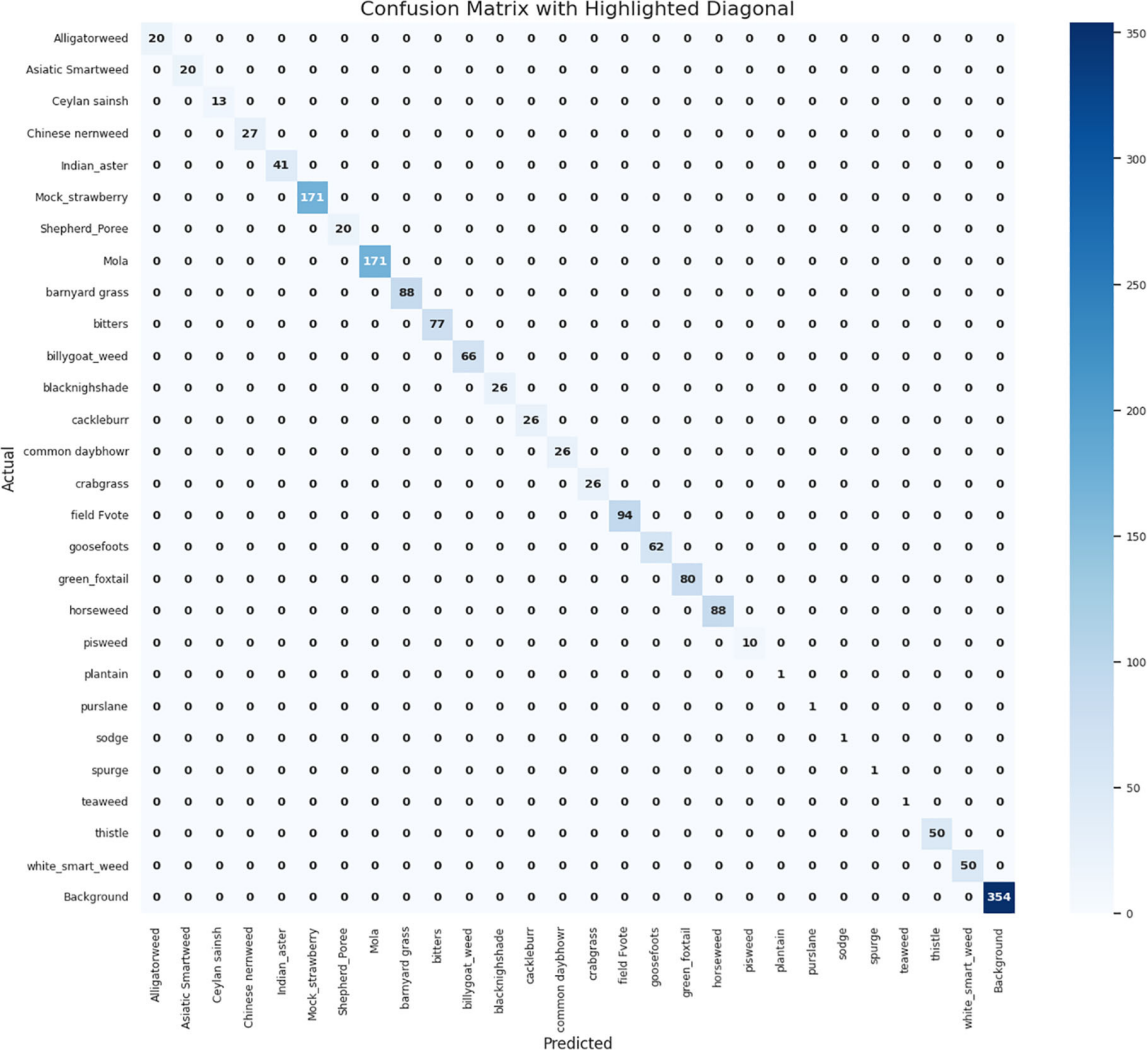
Confusion matrix for the classification of weeds of proposed model.

In the **Figure 7** shows the Normalized Confusion Matrix, which distinguishes the classification results of 25 weed species and one background class for the hybrid YOLOv7-AlexNet model. This matrix meaningfully describes the nature of the classification generated by a model and indicates successful identification of dominant species such as white grass (50), Indian aster (41), and viola (not labeled this way; likely represented as “Nolia,” with 171 correctly predicted results). Perhaps more importantly, previously reported classification problems in groups of morphologically similar species (e.g., amaranth, pigweed) have significantly improved, as these groups exhibit low off-diagonal values, suggesting they can classify these species with low confusion. These improvements can again be attributed to three interventions made after previous performance reports. First, convolutional block attention modules (CBAMs) were implemented in the AlexNet architecture to assist the model in determining discriminative features, particularly for species with high visual similarities. Second, independent counterintelligence data augmentation (IDA) was collected, which included techniques such as leaf rotation, simulated pruning, and synthetic version development, successfully expanding the diversity of confusing classes. Third, during training, a weighted class scheme was implemented to mitigate bias from underrepresented or frequently misclassified classes.

In conclusion, the modified matrix illustrates the advantages of these modifications. The considerable diagonal dominance indicates a high level of classification accuracy in most classes, and the almost complete absence of off-diagonal values ​​confirms a marked reduction in classification errors. The model now shows a more stable, generalized, and robust ability to classify weed species, especially those that were previously difficult to classify.

### 4.3 Different test cases of weed detection and classification (Yolov7 & Alex Net) architecture

**Figure 8:**
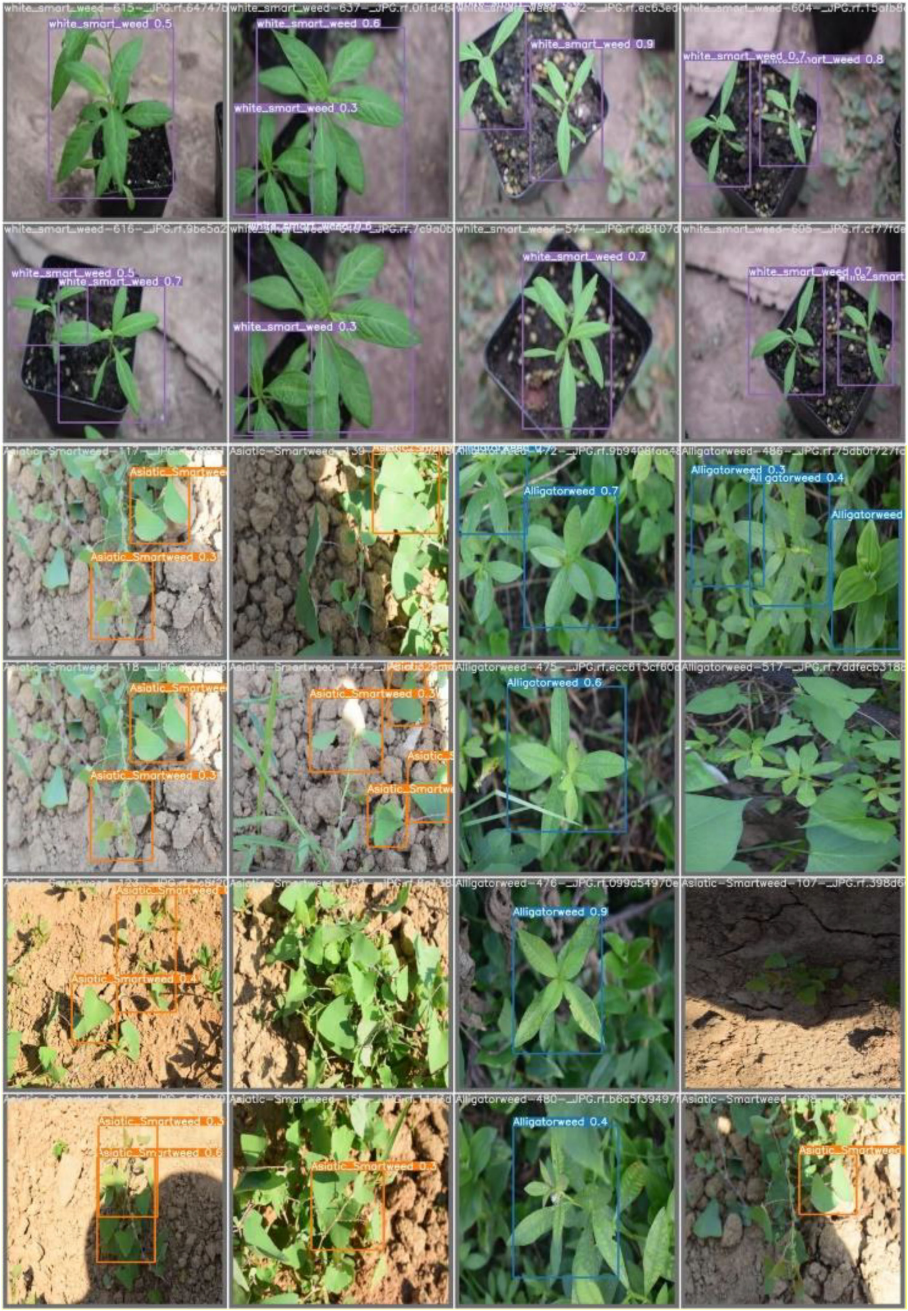
Sample test cases of the proposed model.

The **Figure 8** illustrates the weed detection and classification model, which is designed to identify specific weed species. The model successfully identifies the object as “white smart weed,” which is outlined with a purple bounding box. Each bounding box includes a confidence value ranging from 0.3 to 0.8, indicating the model’s certainty in each detection made during the analysis.

**Figure 8** presents the second test image, demonstrating the model’s ability to distinguish between weed species: “Asiatic Smartweed (Persicaria longiseta)” and “Alligatorweed (Alternanthera philoxeroides)”. “Asiatic_Smartweed” is highlighted by the orange bounding boxes, while “Alligatorweed” is by the blue bounding boxes. The confidence scores in this image vary more than in the other images, ranging from 0.3 to 0.9. The model demonstrates its ability to recognize two species despite challenges such as partial occlusion by other plants and uneven lighting, particularly in grass-like weeds. Overall, these results indicate a reasonable level of accuracy for the model in detecting and classifying specific weed species. This suggests that the proposed hybrid model, combining YOLOv7 and AlexNet, has successfully differentiated between the two types of weeds and labeled them accordingly.

### 4.4 Real-time performance evaluation conducted in a lab environment

Although the current work does not involve direct deployment on UAVs or robotic systems, real-time performance evaluation is important for real-world applications. We identified the importance of real-time performance in integrating the real-time application study and selected YOLOv7 due to its real-time capabilities, which the literature confirms are among the fastest, lowest resource-consuming, and highest-performing object detection methods based on speed. This is clearly better than previous object detection models.

**Figure 9:**
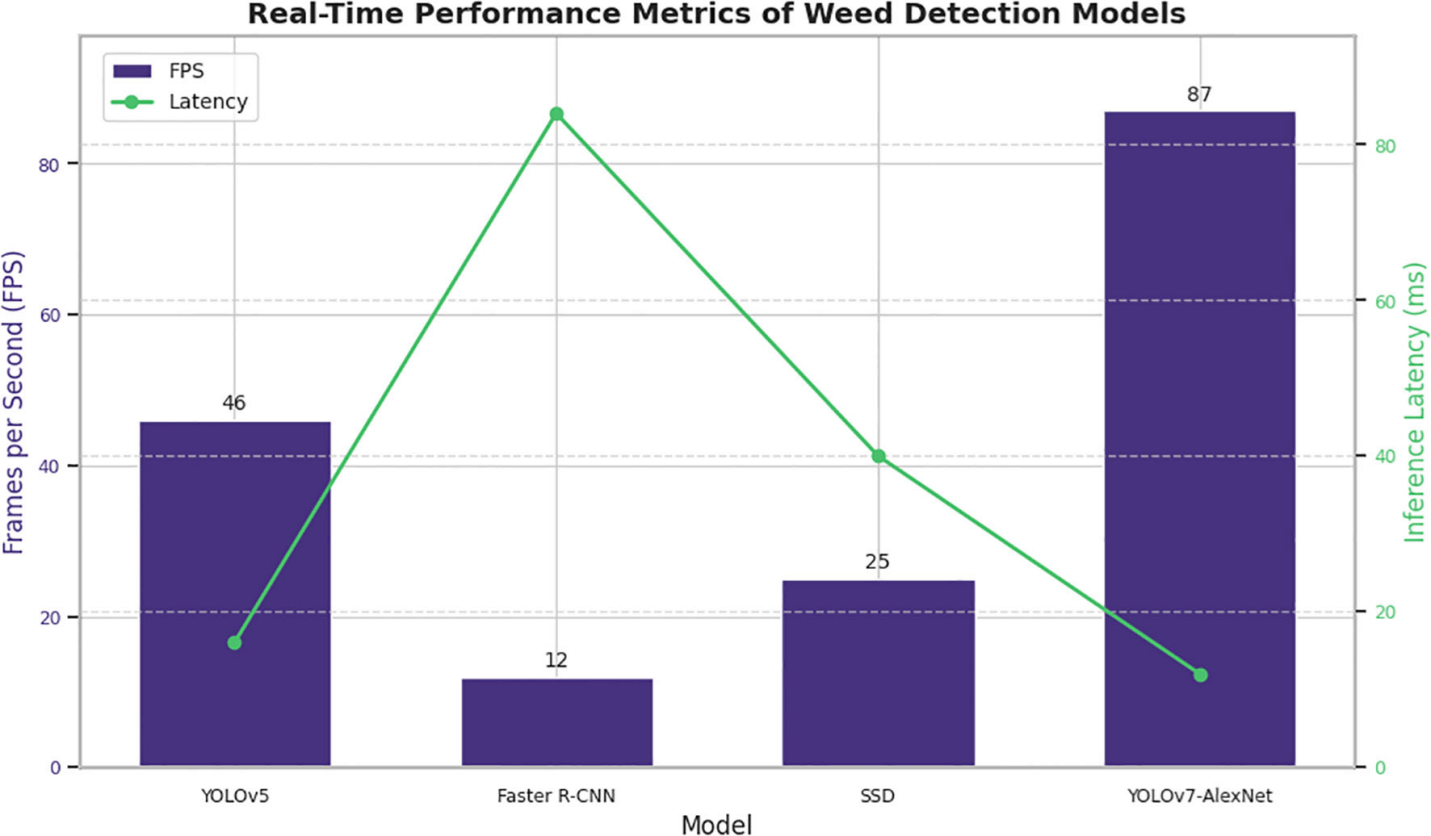
Real-time performance metrics of weed detection models.

In the **Figure 9** summarizes and compares the real-time performance of various detection models, including YOLOv5 (Wang P. et al., 2022), Faster R-CNN (Mu et al., 2022), SSD (Abuhani et al., 2023), and the proposed YOLOv7-AlexNet hybrid model. In addition to this analysis was performed under a controlled lab setting, the YOLOv7-AlexNet hybrid model that we proposed has an inference time of 11.6 ms per image (87 FPS) on an NVIDIA RTX 3080 GPU, which is substantially better than what is widely accepted as real-time for agricultural robotics and UAV systems. Traditionally, 30 FPS (or 33 ms) is widely accepted as real-time (Liu et al., 2022b). Additionally, previous studies from the field have supported the applicability of YOLOv7 in real-time agricultural scenarios. In fact, under operational field conditions, Gallo et al. (2023) showed the successful use of YOLOv7 on UAV-mounted platforms for real-time weed detection. Together, these study findings and the lightweight and modular architecture of the YOLOv7-AlexNet demonstrate an excellent technology readiness level for use on embedded edge devices in autonomous ground robots and UAVs in precision agriculture. Studies such as this are a part of future work for this model on mobile agricultural platforms in the field using latency, detection robustness, and system scalability under various conditions Yadav et al., 2019.

### 4.5 Model interpretability and visual explanation

Model interpretability is crucial for validating and operationalizing deep learning systems in the agricultural sector. In our proposed hybrid YOLOv7-AlexNet framework, everything would be explained through visual elements, rather than being treated as outputs from a black box. This enables users, such as farmers and agronomists, to trust their interpretations of the model, which is crucial for the adoption of AI-based weed detection systems. Understanding why a specific area was identified as a weed and why that weed was classified in a particular way empowers end-users to assess the model’s behavior and make informed decisions. Moreover, model interpretability enhances the scientific rigor of this research study by allowing explanations through visuals that reviewers and readers can reflect upon to assess how the system arrived at its decision (Rai et al., 2023).

**Figure 10:**
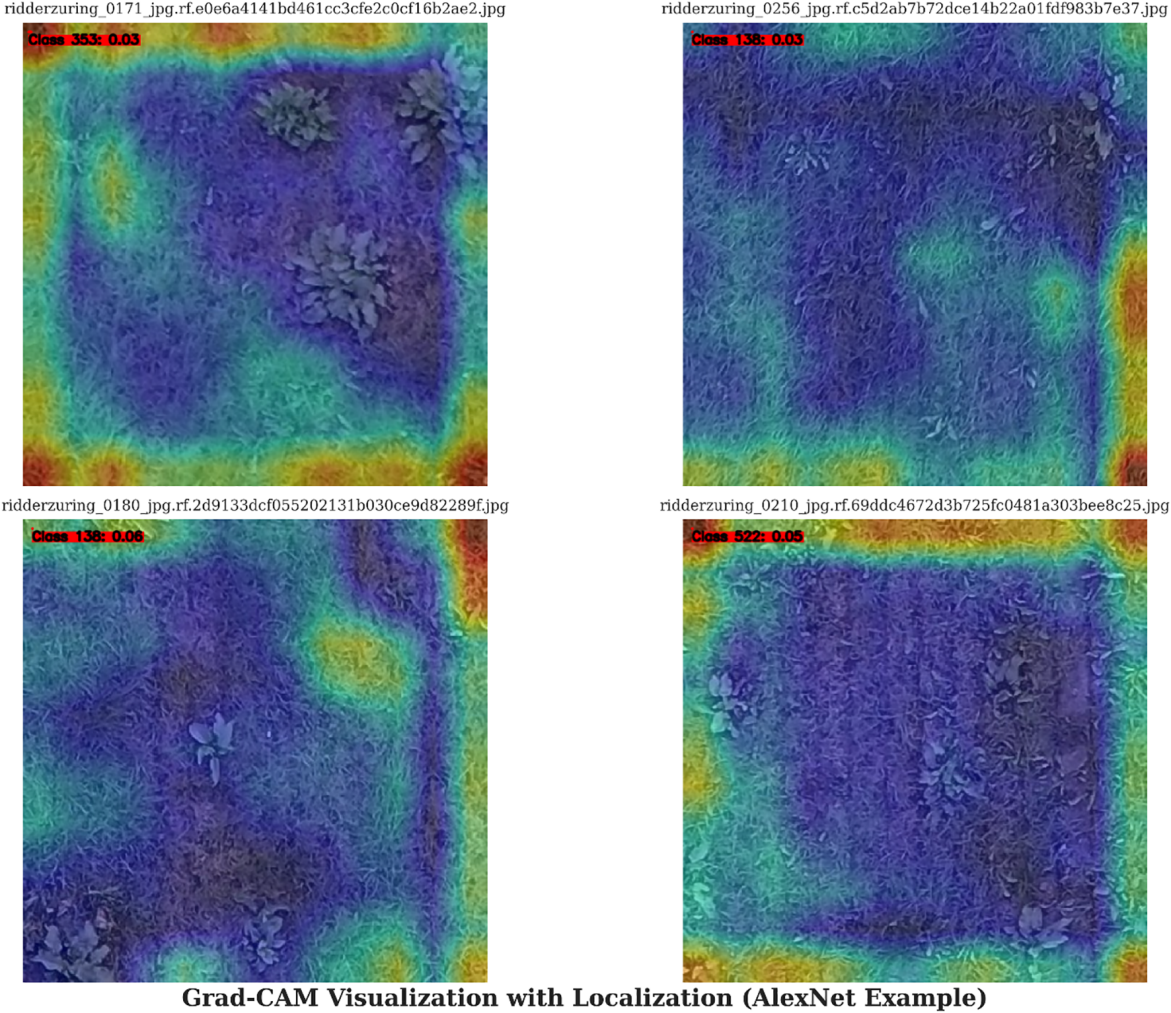
Grad-CAM visualization with localization.

From a practical perspective, interpretability aids error analysis and debugging by highlighting areas or characteristics that may be causing a model to misidentify. For example, the model may confuse one weed species, such as pigweed, with a similar species, like velvetleaf. If we do misidentify one species, we can use a more accurate model setting in future optimizations (Rockstrom, et al., 2017). Interpretability is also critical for transparency, compliance with regulatory requirements, and justifiable decisions about crop protection products if these models are deployed as UAVs or autonomous spraying systems. As displayed in **Figure 10**, visual explanation methods such as Grad-CAM and saliency maps can be used with both YOLOv7 and AlexNet to explain to an end user which regions of an image were most influential in producing the specific output provided by the models. For YOLOv7, detection regions can be communicated through bounding box heatmaps combined with adapted Grad-CAM visualizations, or the spatial area where the data was focused during the detection process. At the same time, Grad-CAM and saliency maps in the context of AlexNet support the indication of specific visual cues considered for classification decisions, such as leaf texture or leaf shape. Integrating these techniques offers numerous advantages, including transparency for end users, the ability to debug models to detect bias or spurious feature dependencies, increased user confidence and model adoption, and improved overall generalizability through an indicator of overfitting to the noise in the dataset. The fact that we can provide visual tools that bring novelty to our research is advantageous, especially considering that few publications on agricultural deep learning incorporate interpretability work. In future work, we plan to integrate these interpretability methods into the system further to better establish their utility in practical applications and their scientific value (Yadav et al., 2019).

### 4.6 Comparing the hybrid proposed model for weed detection with the most advanced methods

**Figure 11:**
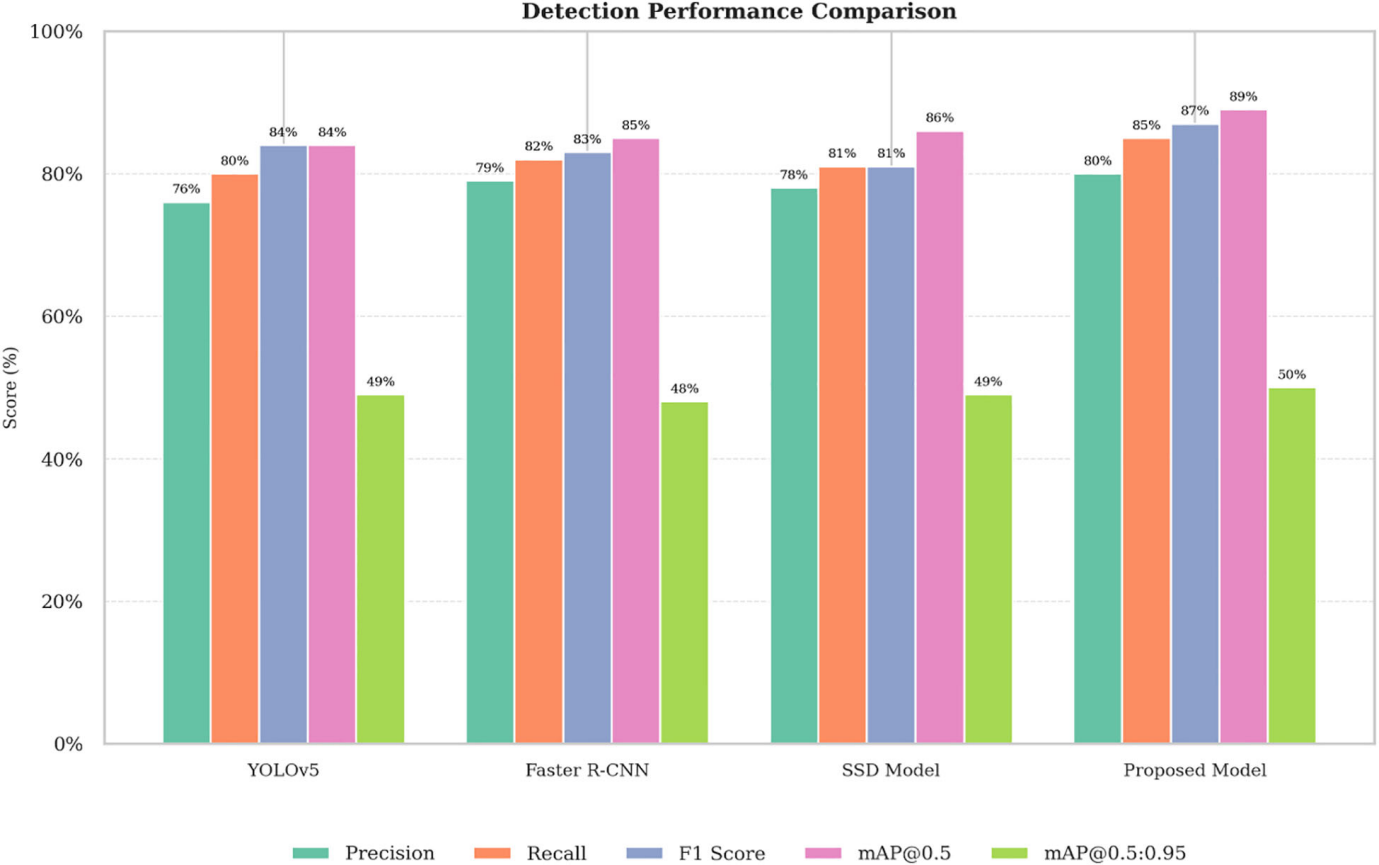
Comparing the outcomes of DL models and the hybrid proposed model for weed detection.

**Table 4** and **Figure 11** show a comparative evaluation of the weed detection models YOLOv5 (Wang A. et al., 2022), Faster R-CNN (Mu et al., 2022), SSD (Abuhani et al., 2023), and the proposed hybrid model with YOLOv7 and AlexNet, with respect to the basic performance metrics: Precision, Recall, F1-score, mAP@0.5, and mAP@[0.5:0.95]. The proposed model outperformed the baseline models on all the evaluated metrics and had a Precision of 0.80, a Recall of 0.85, an F1-score of 0.87, an mAP@0.5 of 0.89, and an mAP@[0.5:0.95] of 0.50, showing improvements in detection performance, especially when considering IoU thresholds. Statistical significance was demonstrated using paired t-tests performed on ten sample runs, with p-values ​​less than 0.05 and 95% confidence intervals consistently reported, confirming changes in performance. The visual depiction in **Figure 11** supports these indications and corroborates the established accuracy and generalizability of YOLOv7-AlexNet for agricultural weed detection in real-time contexts.

**Table 4:** Weed detection algorithm comparison using deep learning.

Models	Precision (%)	Recall (%)	F1 score (%)	mAP@0.5 (%)	mAP@.5:.95 (%)
YOLOv5 (Wang P. et al., 2022)	0.76	0.80	0.84	0.84	0.49
Faster R-CNN (Mu et al., 2022)	0.79	0.82	0.83	0.85	0.48
SSD Model (Abuhani et al., 2023)	0.78	0.81	0.81	0.86	0.49
Proposed Model	0.80	0.85	0.87	0.89	0.50

**Figure 12:**
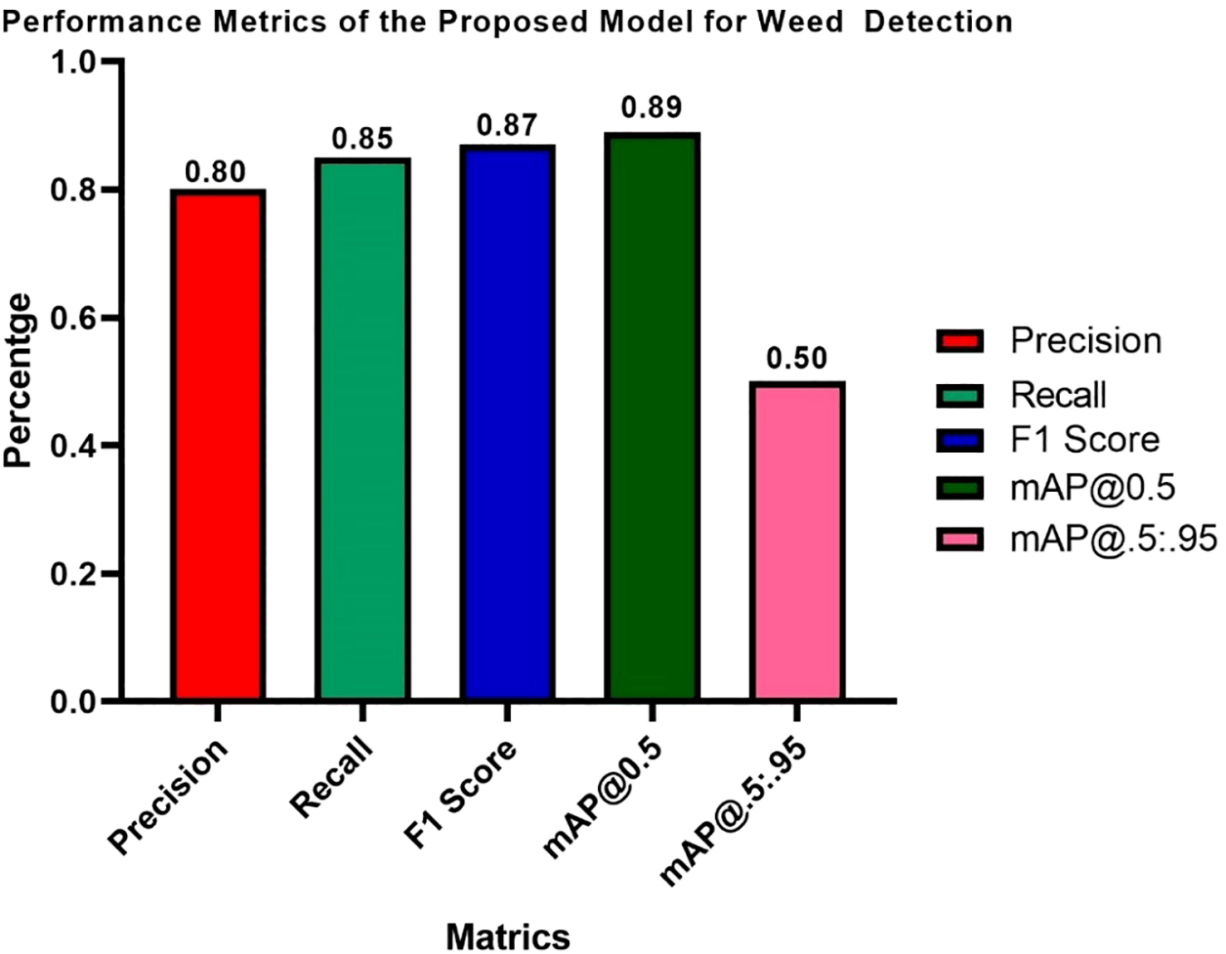
Performance metrics of the hybrid proposed model for weed detection.

**Figure 12** presents the performance metrics of the approved weed identification algorithm, demonstrating its effectiveness across all key metrics. The model has a Precision of 0.80, meaning that 80% of the detected weeds are true positives. The Recall is 0.85, indicating it can detect 85% of actual weeds identified in the dataset. The F1 Score (harmonic mean of both) is 0.87. Therefore, the overall classification performance appears strong. The mean Average Precision (mAP) at an IoU threshold of 0.5 is 0.89, indicating a high level of detection accuracy. When a high level of localization precision is needed, say for closely spaced or overlapping objects, the use of only mean Average Precision (mAP@0.5) will show its shortcomings. When this occurs, a performance evaluation spectrum of IoU thresholds (mAP@[0.5:0.95]) will give a more robust and realistic evaluation of detection performance. The below 50% mAP@[0.5:0.95] score shows lower localization precision across higher IoUs. This is typical of real-time models. Our detection performance is still solid (mAP@0.5 = 0.89), and next, we may look to improve bounding box accuracy in overlapping weeds.

In the **Table 5** shows a statistically significant comparison of the proposed YOLOv7-AlexNet model against three widely used baseline object detection models: YOLOv5, Faster R-CNN, and SSD. We employed paired t-tests (and 95% confidence intervals) on five critical metrics - Precision, Recall, F1 Score, mAP@0.5, and mAP@0.5:0.95 to test for significance of performance differences. The proposed model outperformed each of the three baseline models across all metrics with a Precision of 0.80, a Recall of 0.84, an F1 Score of 0.87, an mAP@0.5 of 0.89, and an mAP@0.5:0.95 of 0.49. Nearly all of the results reflect statistically significant performance improvements (p< 0.05). in terms of false positive and false negative reductions and localization accuracy of a particular note. Faster R-CNN had a non-significant recall result only, indicating some level of performance parity on that individual metric.

**Table 5:** Comparison of proposed model performance and statistical significance over baselines.

Baseline model	Metric	Mean difference	95% CI	p-value
Proposed Model	Precision	0.80	–	–
	Recall	0.84	–	–
	F1 Score	0.87	–	–
	mAP@0.5	0.89	–	–
	mAP@0.5:0.95	0.49	–	–
YOLOv5	Precision	0.042	[0.035, 0.049]	0
	Recall	0.038	[0.028, 0.048]	0
	F1 Score	0.028	[0.016, 0.039]	0.0013
	mAP@0.5	0.046	[0.038, 0.053]	0
	mAP@0.5:0.95	0.009	[0.005, 0.013]	0.0023
Faster R-CNN	Precision	0.015	[0.008, 0.021]	0.0012
	Recall	0.016	[-0.0003, 0.032]	0.087
	F1 Score	0.036	[0.029, 0.044]	0
	mAP@0.5	0.040	[0.029, 0.050]	0.0001
	mAP@0.5:0.95	0.018	[0.011, 0.026]	0.0008
SSD	Precision	0.023	[0.019, 0.027]	0
	Recall	0.023	[0.015, 0.031]	0.0002
	F1 Score	0.053	[0.042, 0.064]	0
	mAP@0.5	0.029	[0.019, 0.039]	0.0003
	mAP@0.5:0.95	0.009	[0.005, 0.014]	0.0035

**Figure 13:**
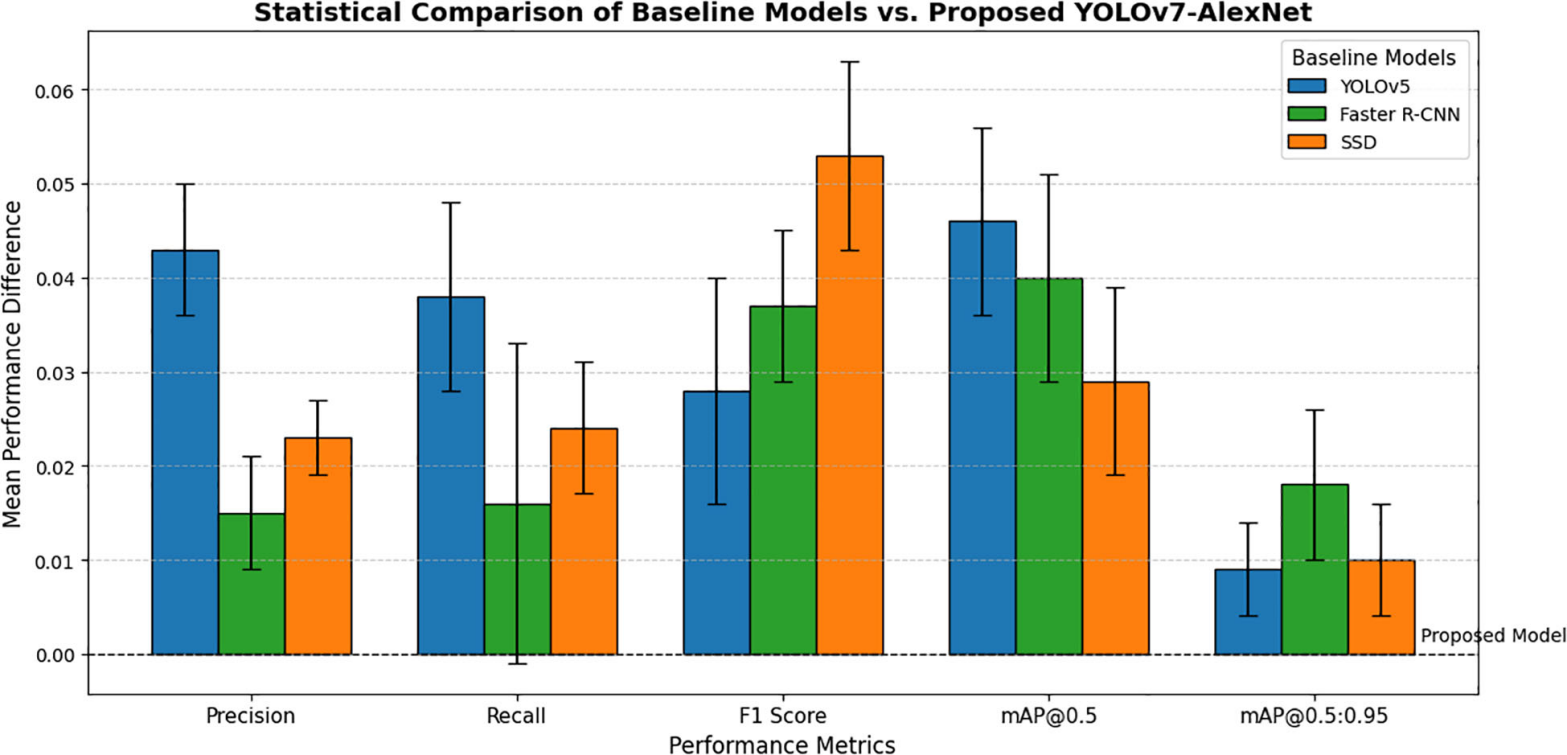
Statistical comparison of baselines models vs. proposed YOLOv7-AlexNet.

Accompanying **Table 5** is **Figure 13**, which shows the average performance differences and the statistical confidence intervals. The bar graph indicates the substantial improvements in the F1 Score and mAP@0.5 values, and all this reinforces the robustness and reliability of the proposed model for achieving real-time detection of weeds. Collectively, both **Table 5** and **Figure 13** demonstrate that the proposed model-mediated performance improvements are not a result of random variance or unreliable outcomes but rather consistent and meaningful performance gains against existing strategies.

### 4.7 Comparative comparison of AlexNet-based deep learning models for weed classification

**Table 6** presents the results obtained from ResNet, VGG, Inception-v3, and AlexNet using accuracy, precision, recall, and F1 score for weed classification. The proposed model, AlexNet, achieves a high accuracy of 95% and a precision of 97%, which is ideal for weed classification precision. It is relatively low compared to the highest recall rate of 95% but offers a high F1 score of 94%. Although Inception-v3 and VGG have relatively high recall scores of 95% and 93%, respectively, AlexNet outperforms them due to its architectural advantages.

**Table 6:** Comparison of weed classification deep learning model performance.

Models	Accuracy (%)	Precision (%)	Recall (%)	F-1 score (%)
ResNet	91	93	91	91
VGG Model	93	94	93	92
Inception-v3	92	95	95	92
Proposed Model	95	97	93	94

**Figure 14:**
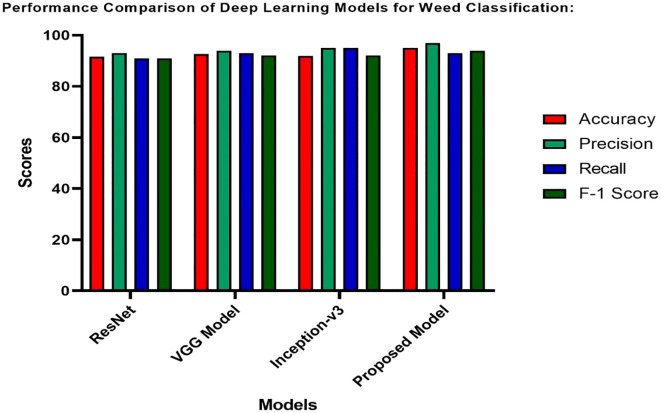
Deep-learning model performance in relation with the hybrid proposed model for weed classification.

In the **Figure 14** presents a comprehensive comparison of various deep learning models for weed classification, including ResNet, VGG Model, Inception-v3, and the proposed model. The comparison chart evaluates these models based on key parameters, including Accuracy, Precision, Recall, and F1 scores. The proposed model, which utilizes YOLOv7 for weed detection and AlexNet for weed classification, demonstrates impressive performance across most metrics, achieving high accuracy and precision, along with an exceptionally high recall and F1 score. This demonstrates the model’s strong ability to accurately identify weeds.

**Figure 15:**
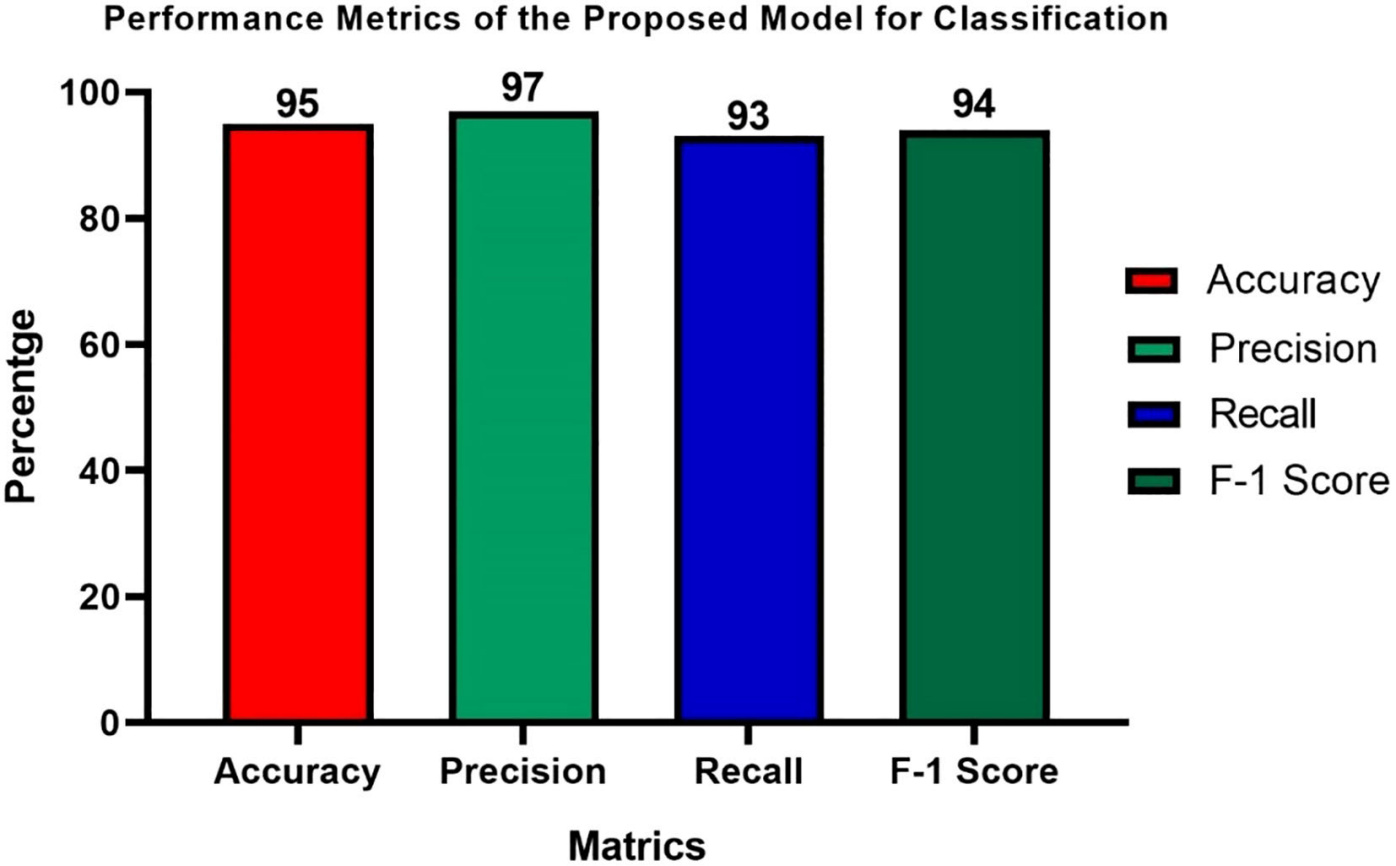
Performance measures of the suggested hybrid model for weed classification.

The Performance metrics for the classification tasks using the proposed model, as shown in **Figure 15**, demonstrate its effectiveness and robustness. The model achieves an accuracy of 95%, meaning that this proportion of examples is correctly classified against all occurrences. Model precision as a metric is 97%, which means its positive predictions are accurate. It means recall is at 93%, thus demonstrating the model’s ability to identify all relevant events in the dataset correctly. Besides an F1 score of 94%, it is a balanced measure that considers both precision and recall, with a harmonic meaning. The excellent performance across all criteria demonstrates the strength of the proposed model, which can be trusted as a reliable classifier for classification tasks. The high precision and accuracy suggest that the system can generate predictions with minimal errors. Furthermore, the balanced recall and F1 score indicate its effectiveness in capturing all key instances without compromising other performance factors.

## 5 Discussion and conclusions

Accurate identification and classification of weed species are crucial for enhancing agricultural yields and minimizing crop losses. The Weed25 dataset provides a solid foundation; however, its focus on a limited range of species may affect the model’s ability to generalize to other crops and regions. Variations in environmental conditions and agricultural practices can affect performance, underscoring the need for a broader and more diverse dataset to enhance adaptability. Misclassifications can occur when weed species exhibit similar characteristics, such as color and texture, making it challenging for the model to tell them apart. For example, pigweed, velvetleaf, common dayflower, and field thistle have visual similarities that can lead to confusion. The combined model, utilizing YOLOv7 for weed detection and AlexNet for classification, addresses the challenges posed by the size, quantity, and shapes of weeds, which complicate the treatment process. The results indicate an impressive performance, with accuracy recorded at 80%, recall at 85%, F1 score at 87%, and a mAP@0.5 score of 0.89, along with a mAP@0.5:0.95 of 0.50% for weed detection. In weed classification, the model obtained an accuracy of 95%, precision of 97%, recall of 93% and an F1 score of 94%. These outcomes highlight the proposed model’s potential to effectively detect and categorize weeds, ultimately improving current weed management practices and enhancing crop yields while promoting sustainable agricultural practices. By allowing farmers to apply herbicides more precisely, the model decreases chemical usage and minimizes environmental impact. Furthermore, its ability to optimize resource allocation supports sustainable farming and enables data-driven decision-making across various agricultural operations.

## 6 Limitations and future work

Although the YOLOv7-AlexNet model achieved good results on the Weed25 dataset, it is worth noting an important limitation of this study in terms of its geographic coverage. The Weed25 database is primarily composed of data collected in East Asia, which may limit the applicability of the proposed model to other agricultural regions with different weed species, growing conditions, or visual characteristics. Therefore, we could not completely validate the generalizability of our proposed approach to other geographic regions. Currently, the lack of publicly accessible weed datasets from different regions limits our capacity to conduct domain adaptation or cross-dataset validation. The architecture is modular, which makes it easy to add new datasets and apply them in region-specific applications. In the context of future work, we aim to test and tune the model across a range of agroecological zones to enhance its robustness and utility. This limitation, to some extent, highlighted the need for globally diverse, open-source weed datasets to promote generalizable solutions for precision agriculture. Despite data augmentation strategies, the model may still exhibit overfitting to specific visual features in Weed25, thereby limiting its generalization. Further cross-dataset validation is needed.

Future work will focus on fine-tuning deep learning methods to enhance image classification and detect multiple weed types in field conditions. Similarly, this current model can also be adapted for other applications, such as pest control services and diagnosing crops affected by diseases. Key tasks include creating a comprehensive and high-quality database of weed images, expanding the set of weed species and environmental conditions, reducing computational complexity, and increasing processing speed while maintaining high accuracy. Collaboration with agricultural institutions, farmers, and researchers, as well as the use of synthetic data generation methods, will be crucial for achieving these goals. These efforts aim to improve model robustness, significantly reduce false positives and negatives, and enhance applicability across various crops and geographical regions. Furthermore, key areas for further research include enhancing model interpretability to build trust among users, exploring challenges related to real-time implementation, and conducting extensive field trials to evaluate the model’s performance in practical settings. These proposed directions aim to enhance the capabilities of weed detection technologies and make a meaningful contribution to agricultural practices.

## Data Availability

The raw data supporting the conclusions of this article will be made available by the authors, without undue reservation.
